# Estimation of gender-specific connectional brain templates using joint multi-view cortical morphological network integration

**DOI:** 10.1007/s11682-020-00404-5

**Published:** 2020-10-21

**Authors:** Nada Chaari, Hatice Camgöz Akdağ, Islem Rekik

**Affiliations:** 1 BASIRA Lab, Faculty of Computer and Informatics, Istanbul Technical University, Istanbul, Turkey; 2 Faculty of Management, Istanbul Technical University, Istanbul, Turkey; 3 Computing, School of Science and Engineering, University of Dundee, Dundee, UK

**Keywords:** Cortical morphological networks, Gender differences, Connectional brain template estimation, Multi-view clustering, Population-driven connectome, Brain connectome atlas learning

## Abstract

The estimation of a connectional brain template (CBT) integrating a population of brain networks while capturing shared and differential connectional patterns across individuals remains unexplored in gender fingerprinting. This paper presents the first study to estimate gender-specific CBTs using multi-view cortical morphological networks (CMNs) estimated from conventional T1-weighted magnetic resonance imaging (MRI). Specifically, each CMN view is derived from a specific cortical attribute (e.g. thickness), encoded in a network quantifying the dissimilarity in morphology between pairs of cortical brain regions. To this aim, we propose Multi-View Clustering and Fusion Network (MVCF-Net), a novel multi-view network fusion method, which can jointly identify consistent and differential clusters of multi-view datasets in order to capture simultaneously similar and distinct connectional traits of samples. Our MVCF-Net method estimates a representative and well-centered CBTs for male and female populations, independently, to eventually identify their fingerprinting regions of interest (ROIs) in four main steps. *First*, we perform multi-view network clustering model based on manifold optimization which groups CMNs into shared and differential clusters while preserving their alignment across views. *Second*, for each view, we linearly fuse CMNs belonging to each cluster, producing local CBTs. *Third*, for each cluster, we non-linearly integrate the local CBTs across views, producing a cluster-specific CBT. Finally, by linearly fusing the cluster-specific centers we estimate a final CBT of the input population. MVCF-Net produced the most centered and representative CBTs for male and female populations and identified the most discriminative ROIs marking gender differences. The most two gender-discriminative ROIs involved the lateral occipital cortex and pars opercularis in the left hemisphere and the middle temporal gyrus and lingual gyrus in the right hemisphere.

## Introduction

Several human neuroimaging studies have been conducted to analyze brain connectivity between regions with respect to gender differences providing fundamental insights into the organization and integration of brain networks in male and female populations (Ingalhalikar et al. 2014; Jiang et al. 2019). In particular, brain connectivity models interactions between different regions, which can be leveraged to investigate gender fingerprinting. Gender differences can be identified using functional connectivity and structural connectivity, derived from functional magnetic resonance imaging (fMRI) and diffusion weighted imaging (DWI) respectively (Tyan et al. 2017; Jiang et al. 2019; Dadashkarimi et al. 2019). By explicitly deriving structural and functional brain connectivity from functional and diffusion-weighted magnetic resonance imaging (fMRI and dMRI), network analysis presents a powerful tool for exploring structural–functional connectivity relationships (Zhou et al. 2006; Honey et al. 2007, 2009) and revealing the causative linkage between connectivity changes and task performance across genders (Bolla et al. 2004).

Several studies (Spelke 2005; Koch et al. 2007; Keller and Menon 2009; Derntl et al. 2010) on sex differences revealed contrasting activation patterns in cognitive abilities, behaviors and emotions between male and female brains. Such studies provide a better understanding of learning processes, language development, and progression of neurologically-based diseases such as autism spectrum disorder and depression (Zaidi 2010; Werling and Geschwind 2013) across genders. What’s more, early prediction, risk, and protective factors of brain disorders can be captured, as well as personalized treatments for male and female populations can be designed. Although fMRI and dMRI neuroimaging modalities allowed the discovery of predictive brain connections fingerprinting gender differences, they may have a few limitations. On the one hand, functional MRI can produce spurious and noisy connectomes due to the low signal-to-noise ratio induced by non-neural noise (Soussia and Rekik 2018). On the other hand, diffusion MRI can produce biased and largely variable structural connectomes depending on the employed fiber tractography method; a fact supported by a recent study (Petrov et al. 2017) which evaluated 35 methods to generate structural connectomes and showed that how variations in diffusion MRI pre-processing steps affect network reliability and its ability to classify subjects remains opaque.

To circumvent the limitations of these neuroimaging modalities, recent studies have explored an alternative brain network representation, a cortical morphological network (CMN) constructed from structural T1-w MRI. The main idea is to build a network based on morphological connections of the cortical surface derived from a unique cortical attribute such as sulcal depth or cortical thickness. Specifically, CMNs model the relationship in morphology between different cortical regions quantified using specific cortical measurements. For instance, CMNs were investigated in neurodegenerative disorders (Mahjoub et al. 2018; Lisowska et al. 2019) as well as in neuropsychiatric disorders (Soussia and Rekik 2018; Georges et al. 2020). (Nebli and Rekik 2019) presented the first study on gender differences using CMNs of healthy subjects. This work leverages a feature selection method to find the most discriminative morphological connections between male and female cortices using different cortical attributes. Although compelling, this study might discard some of the important connectional features (CFs) in revealing the gender-specific brain connectional map. In fact, the utilized feature selection method selects only the important CFs and eliminates others which can lead to losing rich information when creating holistic maps of the male and female multi-view CMNs.

On the other hand, the concept of connectional brain template (CBT) comes in to normalize a set of multi-view brain networks, while considering all connectivities to enable the integration of complementary information and the production of a representative `average’ of a given population. Hence, the estimation of a CBT provides an excellent tool for mapping human psychological behavior and cognitive functions, by providing a representative and holistic connectional map of a set of multi-view brain networks. As integral and normalized representations of the multi-view brain connectivity, CBTs estimated for each gender, can hence help spot out different connectional patterns between the male and female brains. (Rekik et al. 2017) presents the first study on the estimation of a centered CBT using a population of brain networks based on a diffusive-shrinking graph technique. However, this work was limited to handling single-view networks, thereby overlooking the complementary and richness of multi-view brain networks populations, where each individual is represented by a set of brain networks (i.e., views). (Dhifallah et al. 2019) generalized this concept to multi-view brain networks for a more holistic and integral mapping of brain connectivity by first non-linearly fusing multiview brain networks for each individual in the population, and secondly by clustering the fused networks and integrating them within each cluster, and finally by averaging the obtained centers of all clusters. Despite its promising performance, this study has a major drawback which is clustering the samples without considering their heterogeneity across views which fails to simultaneously capture the distinct and the shared population-specific traits.

To address these limitations, we propose MVCF-Net, a novel multi-view network brain connectivity clustering-fusion method that estimates a representative and centered CBTs for a given population, with application to gender fingerprinting. Our method is rooted in the identification of consistent and differential clusters across brain views to generate a representative and well-centered CBT for a given population and to reduce subject inter-variability. To this aim, first, we leverage multi-view network clustering model based on manifold optimization method (Yu et al. 2019), which performs clustering across data views. Thus, similar connectional traits and distinct connectional traits of samples within and across clusters in different views can be identified in an unsupervised way (Yu et al. 2019). Second, for each view, we linearly average the CMNs of the subjects within each cluster, so that each cluster is represented by a local CBT. Third, for each aligned cluster, we nonlinearly integrate its local CBTs across views into a cluster-specific CBT. Finally, we linearly fuse the cluster-specific CBTs to estimate the final CBT representing a given population. The estimated CBT captures both shared and distinct traits of a population. Ultimately, by simply comparing the CBTs derived from female and male populations, respectively, we spot out gender connectional differences. We demonstrate that the resulting multi-view population-driven CBTs by MVCF-Net fulfill the following criteria: (i) they are well-centered and they achieve the minimum Frobenius distance to all brain views and all subjects in a given population, and (ii) they can effectively differentiate gender fingerprints by capturing the most discriminative brain connections regions between male and female cortices.

## Material

### Dataset and data prepossessing steps

We evaluate our proposed MVCF-Net method using the brain genomics superstruct project (GSP) dataset (Buckner et al. 2012; Holmes et al. 2015) detailed in Table 1. The dataset consists of 698 healthy candidates split in two populations: 308 subjects are males and 390 subjects are females, and none of them carry any sign of brain disorders or had any history of mental disease. Each subject is represented with structural T1-w MR image which undergoes preprocessing steps such motion and topology correction, T1-w intensity normalization and segmentation of the subcortical white and deep grey matters volumetric structures (Nebli and Rekik 2019). Then, we leverage the reconstruction of the right and the left cortical hemispheres (RH and LH) for each subject (Nebli and Rekik 2019). Next, we partition each hemisphere into N_r_ = 35 cortical regions of interest (ROIs) using Desikan-Killiany Atlas (Fischl et al. 2004) and FreeSurfer (Fischl 2012). Finally, for each subject n and for each hemisphere, we define M = 4 networks ${\left\{{\mathbf{V}}_{\mathrm{n}}^{\mathrm{m}}\right\}}_{m=1}^M$, where each is represented by a cortical morphological network (CMN): ${\mathbf{V}}_{\mathrm{n}}^1$ indicates the maximum principal curvature brain view, ${\mathbf{V}}_{\mathrm{n}}^2$ marks the mean cortical thickness brain view, ${\mathbf{V}}_{\mathrm{n}}^3$ is generated using the mean sulcal depth, and ${\mathbf{V}}_{\mathrm{n}}^4$ is derived from the mean cortical curvature. Brain morphological networks are constructed separately for the left and the right hemispheres, and they are investigated independently as we aimed in this study to overlook morphological connections that can be “biased” by the brain hemispheric asymmetry (Witelson and Pallie 1973; Wada et al. 1975). Combining them also prevents the loss of insightful information on how gender affects each hemisphere independently.

**Table 1:** Data distribution of female/male dataset

Dataset	Male	Female
Number of subjects	308	390
mean ± std. age	21.6 ± 0.9	21.6 ± 0.8

### Cortical morphological network construction

We represent each subject n by a set of M multi-view brain networks, where we hypothesize that brain networks of a single view m lie on a manifold **M**_m_. We denote by M the number of manifolds and $\left\{{\mathbf{V}}_{\mathrm{n}}^1,{\mathbf{V}}_{\mathrm{n}}^2,\dots, {\mathbf{V}}_{\mathrm{n}}^{\mathrm{M}}\right\}$ are the brain networks where ${\mathbf{V}}_{\mathrm{n}}^{\mathrm{m}}$ is the CMN of the m^th^ view representing subject n nested in **M**_m_ manifold. We denote by $\mathfrak{m}$ in *ℝ* the index of a brain network view and by ${\mathrm{V}}_{\mathrm{n}}^{\mathrm{m}}$the brain network of the $\mathfrak{m}$^th^ view for subject n. A view $\mathfrak{m}$ is a description of a single view in the brain network tensor of a single subject (e.g., mean cortical thickness brain view), which is represented by a vectorized brain network lying in a high dimensional space. On the other hand, a view-specific manifold **M**_m_ is a learnable topological space where all m^th^ brain network views of all subjects are nested. In other words, a view-specific manifold **M**_m_ aims to embed brain networks of the m^th^ view of each subject into a low dimensional space. We represent each single-view brain network as a complete graph comprising N_r_ nodes, where each node denotes an ROI in the cortex, and the edge denotes the connection quantifying the dissimilarity strength between two ROIs. The graph can be mathematically encoded in an N_r_ × N_r_ symmetrical matrix ${\mathbf{V}}_{\mathrm{n}}^{\mathrm{m}}$, where each element ${\mathbf{V}}_{\mathrm{n}}^{\mathrm{m}}\left(\mathrm{i},\mathrm{j}\right)$ ∈${\mathbf{V}}_{\mathrm{n}}^{\mathrm{m}}$ measures the interaction or relationship between R_i_ and R_j_. Specifically, we define ${\mathbf{V}}_{\mathrm{n}}^{\mathrm{m}}\left(\mathrm{i},\mathrm{j}\right)$ as the absolute difference between the means of cortical attributes ${\overset{\sim }{\mathrm{mc}}}_i$and ${\overset{\sim }{\mathrm{mc}}}_j$ (i.e. cortical thickness) respectively in regions R_i_ and R_j_: $\left|{\overset{\sim }{\mathrm{mc}}}_i-{\overset{\sim }{\mathrm{mc}}}_j\right|$. #{v ∈ R_i_ } denotes the number of vertices v belonging to ROI R_i_ and mc(v) is the cortical measurement value assigned to vertex v, we compute the average of mc(v) across all vertices v in R_i_ as follows:

1 $$ {\overset{\sim }{\mathrm{mc}}}_i=\frac{1}{\#\left\{\mathrm{v}\in {\mathrm{R}}_{\mathrm{i}}\ \right\}}{\sum}_{\mathrm{v}\in {\mathrm{R}}_{\mathrm{i}}}\mathrm{mc}\left(\mathrm{v}\right)\kern0.5em $$

## Method

### Overview

**Fig. 1:**
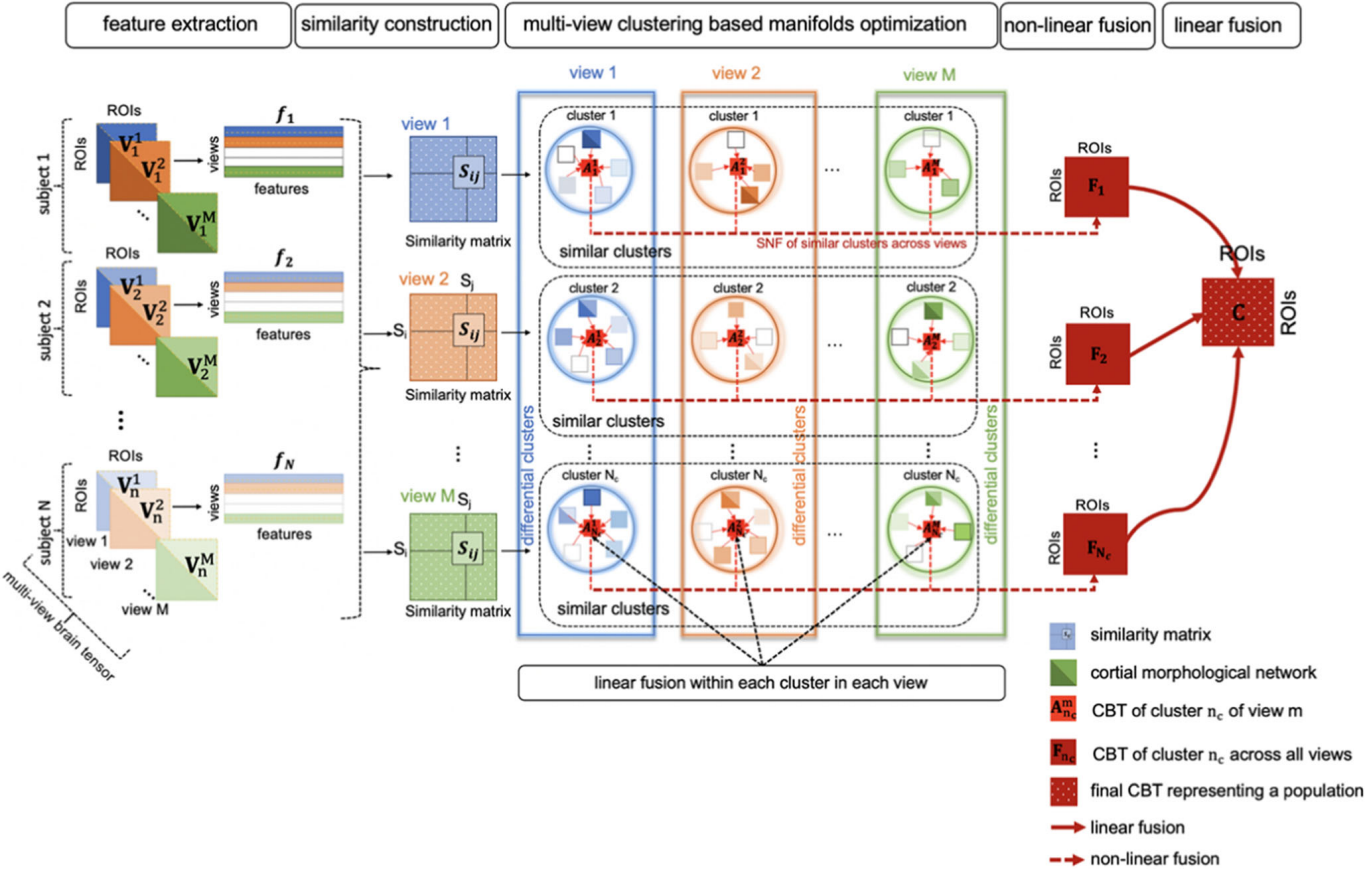
*Pipeline of the proposed MVCF-Net framework for connectional brain template (CBT) estimation using multi-view brain networks.* First, for a given brain network view and for each subject, we extract features by vectorizing the upper off-diagonal part of each brain connectivity matrix. Second, we compute the Euclidian distance between each pair of subjects using their corresponding features vectors to eventually derive a multi-view similarity matrix. Third, we perform multi-view network clustering based on manifold optimization method (Yu et al. 2019) to partition subjects into shared and differential clusters across views. Fourth, we linearly average all brain networks in each cluster as they lie close to each other, producing local CBTs. Next, for each cluster, we nonlinearly fuse its local CBTs across each view using similarity network fusion (SNF) since the local CBTs might lie far from each other. This produces a cluster-specific CBT. Last, we average all cluster-specific CBTs across all clusters, thereby generating the global population CBT

**Fig. 2:**
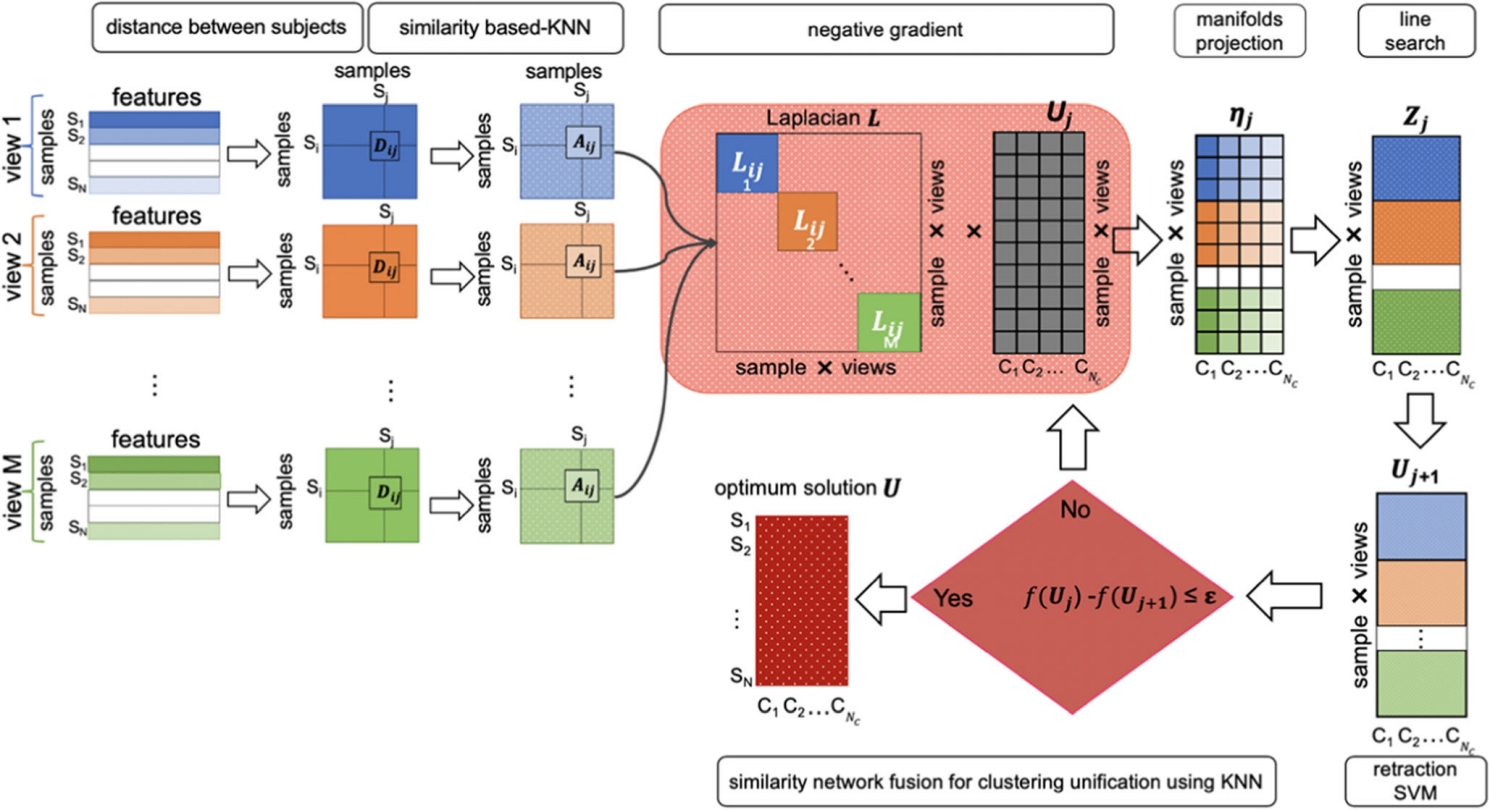
*Multi-view clustering using manifold optimization*. For each view *m* lying on a manifold **M**_m_, first, we calculate pairwise distance matrix between subjects. Second, for each view *m*, we derive the similarity matrices using K-nearest neighbor (KNN) method and compute the Laplacian matrix. Then, for each view *m*, we partition all subjects into clusters while preserving their alignment using multi-view spectral clustering. Thus, both consistent and differential clusters can be identified simultaneously. To do that, we solve the optimization problem for each view: min*trace*(***U***_***m***_^***T***^ ***L U***_***m***_) where **U**_**m**_ is a vector representing the initial partition of N subjects into N_C_ cluster. The optimization process includes three steps: first we project the negative gradient on the tangent vector to the manifold m and we obtain the direction η_**m**_. Second, we update **U**_**m**_ by adding a multiple of this direction to its previous measurement. Third, we retract the new **U**_**m + 1**_ to the manifold using single value decomposition. Finally, as ***U*** converges, we compute k-means clustering to obtain the final label vector partitioning the *N* subjects into N_C_ clusters for each network view

In this section, we detail our joint multi-view network clustering and fusion framework MVCF-Net to estimate a population-based CBT from a set of multi-view CMNs. We illustrate in Figs. 1 and 2 the four proposed steps of MVCF-Net: 1) feature extraction similarity networks construction, 2) multi-view clustering using optimization manifolds, 3) Individual-based non-linear fusion of connectional brain views, and 4) Linear fusion. Furthermore, we detail our evaluation strategies for assessing the representativeness and discriminability of the estimated CBTs as well as the identified of the top discriminative regions of interest differentiating both genders. For easy reference, we summarize the major mathematical notations in Table 2 and detail the steps of the proposed MVCF-Net framework in Algorithm 1.

**Table 2:** Major Mathematical notations used in this paper

Math notation	Dimension	Definition
***M***	*ℕ*	number of views
***m***	*ℕ*	view m
*n*	*ℕ*	subject n
**M**_**m**_	–	manifold of $\mathfrak{m}$^th^ view, 1 ≤ m ≤ M
***N***	*ℕ*	number of subjects in a given population, 1 ≤ n ≤ N
***N***_***r***_	*ℕ*	number of regions of interest in a brain network (ROIs)
**R**_***i***_	–	regions of interest i, 1 ≤ i ≤ N_r_
***N***_***c***_	*ℕ*	number of clusters
***N***_***t***_	*ℕ*	number of iterations in SNF algorithm
***N***_***f***_	*ℕ*	dimension of feature vector ${\mathrm{f}}_{\mathrm{n}}^{\mathrm{m}}$
***K***	*ℕ*	number of folds used for cross-validation partition
***K***_***n***_	*ℕ*	number of the nearest neighbors used for KNN algorithm
${\mathbf{C}}_{{\boldsymbol{n}}_{\mathbf{c}}}^{\mathbf{m}}$	–	cluster *n*_c_ in $\mathfrak{m}$^th^ view, 1 ≤ n_c_ ≤ N_c_
${\overset{\sim }{\boldsymbol{mc}}}_{\boldsymbol{i}}$	*ℝ*	mean cortical attribute of R_*i*_
$\kern0.5em {\mathbf{V}}_{\mathbf{n}}^{\mathbf{m}}$	${\mathbb{R}}^{{\mathrm{N}}_{\mathrm{r}}\times {\mathrm{N}}_{\mathrm{r}}}$	brain network of $\mathfrak{m}$^th^ view for subject n
***D***_***m***_	*ℝ*^N × N^	distance matrix of $\mathfrak{m}$^th^ view
$\kern0.5em {\boldsymbol{f}}_{\mathbf{n}}^{\boldsymbol{m}}$	${\mathbb{R}}^{{\mathrm{N}}_f\times 1}$	feature vector of $\mathfrak{m}$^th^ view for subject n
***S***_***m***_	*ℝ*^N × N^	similarity matrix of $\mathfrak{m}$^th^ view
***W***_***m***_	*ℝ*^N × N^	diagonal matrix of $\mathfrak{m}$^th^ view of matrix **S**_**m**_
***L***_***m***_	*ℝ*^N × N^	Laplacian matrix of $\mathfrak{m}$^th^ view of matrix ***S***_***m***_
***U***_***m***_	${\mathbb{R}}^{\mathrm{N}\times {\mathrm{N}}_c}$	assignment matrix of $\mathfrak{m}$^th^ view of all subjects into N_C_ clusters
η_***m***_	${\mathbb{R}}^{\mathrm{N}\times {\mathrm{N}}_c}$	eigenvector of $\mathfrak{m}$^th^ view of the Laplacian ***L***_***m***_
**V**	${\mathbb{R}}^{{\mathrm{N}}_c\times {\mathrm{N}}_c}$	right singular vectors decomposition of **U**
**W**	${\mathbb{R}}^{\mathrm{N}\times {\mathrm{N}}_c}$	left singular vectors decomposition of **U**
**U**	${\mathbb{R}}^{\left(\mathrm{M}\times \mathrm{N}\right)\times {\mathrm{N}}_c}$	representation of **U**_**m**_in all network views
$\kern0.5em {\boldsymbol{A}}_{{\mathbf{n}}_{\mathbf{c}}}^{\boldsymbol{m}}$	${\mathbb{R}}^{{\mathrm{N}}_{\mathrm{r}}\times {\mathrm{N}}_{\mathrm{r}}}$	estimated CBT of cluster n_c_ in $\mathfrak{m}$^th^ view
$\kern0.5em {\boldsymbol{P}}_{{\mathbf{n}}_{\mathbf{c}}}^{\boldsymbol{m}}$	${\mathbb{R}}^{{\mathrm{N}}_{\mathrm{r}}\times {\mathrm{N}}_{\mathrm{r}}}$	full kernel matrix for $\mathfrak{m}$^th^ view and cluster n_c_
$\kern0.5em {\boldsymbol{S}}_{{\mathbf{n}}_{\mathbf{c}}}^{\boldsymbol{m}}$	${\mathbb{R}}^{{\mathrm{N}}_{\mathrm{r}}\times {\mathrm{N}}_{\mathrm{r}}}$	sparse kernel matrix for $\mathfrak{m}$^th^ view and cluster n_c_
${\boldsymbol{F}}_{{\mathbf{n}}_{\mathbf{c}}}$	${\mathbb{R}}^{{\mathrm{N}}_{\mathrm{r}}\times {\mathrm{N}}_{\mathrm{r}}}$	fused CBT of cluster n_c_ across all views
**C**	${\mathbb{R}}^{{\mathrm{N}}_{\mathrm{r}}\times {\mathrm{N}}_{\mathrm{r}}}$	estimated connectional brain template
***q***	*ℕ*	number of subjects in cluster n_c_
T	*ℝ*	absolute difference matrix between two CBTs
**α**	*ℝ*	discriminative score vector of ROIs distinguishing two groups
***y***^***m***^	*ℕ*	class label vector of all subjects in $\mathfrak{m}$^th^ view
**x**	${\mathbb{R}}^{{\mathrm{N}}_f\times 1}$	weight feature vector of ROIs
***B***	${\mathbb{R}}^{{\mathrm{N}}_{\mathrm{r}}\times {\mathrm{N}}_{\mathrm{r}}}$	discriminative weight matrix of ROIs

*KNN* K-Nearest Neighbors, *SNF* Similarity Network Fusion, *SVD* singular vectors decomposition

### Feature extraction and similarity networks construction

First, for each view m we extract the off-diagonal elements of the upper triangular part of each brain network encoded in a symmetric connectivity matrix to form the feature vector ${\mathrm{f}}_{\mathrm{n}}^{\mathrm{m}}$. The dimension of each feature vector is thus equal to N_f_ = N_r_ × (N_r_ − 1)/2 . Next, for each view m, we define a pairwise distance matrix **D**_m_between subjects, where **D**_m_(i, j) is the Euclidian distance between subject i and subject j using their feature vectors ${\mathbf{f}}_{\mathrm{i}}^{\mathrm{m}}$ and ${\mathbf{f}}_{\mathrm{j}}^{\mathrm{m}}$. We then generate the similarity matrix **S**_m_ based on the distance (i.e., dissimilarity) matrix **D**_m_to capture the similarity strength between each pair of subjects. We denote by **S**_ij_ the similarity value between subjects i and j, where **S**_ij_ approaches zero when i and j are dissimilar.

### Multi-view clustering using optimization manifolds (Step1)

Unlike other methods (Dhifallah et al. 2019) which generate CBTs by directly fusing heterogeneous connectional brain networks of a given population, first, we group subjects into more homogenous clusters by leveraging a multi-view clustering model developed by (Yu et al. 2019), which returns the aligned clusters in each view. Thus, both the consistent clusters and the differential clusters are identified in each view. Specifically, for each manifold **M**_m_, we transform the connectional brain networks to similarity matrices that measure the relation between different subjects. Next, for each view, we partition subjects into aligned clusters by solving an optimization problem using the line-search method and then by applying k-means clustering. While the line-search method returns the assignment of subjects into all clusters for each view, k-means clustering method groups subjects into clusters. Thus, the aligned clusters are identified in each view. We detail these steps in the following part.

First, we construct the diagonal matrix **W**_**m**_ by summing each row of **S**_**m**_ as indicated in Eqs. (2) and (3), then perform spectral clustering to solve the optimization model (Zhang et al. 2015) as follows:

2 $$ {\mathbf{W}}_{\mathrm{m}}={\operatorname{diag}}_{1\le \mathrm{i}\le \mathrm{N}}\left({\mathbf{S}}_{\mathrm{i}}\right) $$

3 $$ \mathrm{where}\kern0.75em {\mathbf{S}}_{\mathrm{i}}=\sum \limits_{\mathrm{j}=1}^{\mathrm{N}}{\mathbf{S}}_{\mathrm{i}\mathrm{j}} $$

4 $$ {\min}_{{\mathrm{U}}_{\mathrm{m}}\in {\mathrm{R}}^{\mathrm{N}\times {\mathrm{N}}_{\mathrm{c}}}}\mathrm{trace}\left({\mathbf{U}}^{\mathrm{T}}\mathbf{LU}\right)\kern0.5em \mathrm{s}.\mathrm{t}.\kern0.5em {\mathbf{U}}^{\mathrm{T}}\mathbf{U}={\mathbf{I}}_{{\mathrm{N}}_{\mathrm{c}}} $$

5 $$ \left\{\begin{array}{c}\mathrm{where}\kern1em \mathbf{L}=\left(\begin{array}{ccc}\begin{array}{cc}{\mathbf{L}}_1& 0\\ {}0& {\mathbf{L}}_2\end{array}& \cdots & \begin{array}{c}0\\ {}0\end{array}\\ {}\vdots \kern2em \vdots & \ddots & 0\\ {}0\kern0.5em 0& \cdots & {\mathbf{L}}_{\mathrm{M}}\end{array}\right)-\left(\begin{array}{ccc}\begin{array}{cc}0& {\mathbf{I}}_{\mathrm{n}}\\ {}{\mathbf{I}}_{\mathrm{n}}& 0\end{array}& \cdots & \begin{array}{c}{\mathbf{I}}_{\mathrm{n}}\\ {}{\mathbf{I}}_{\mathrm{n}}\end{array}\\ {}\vdots \kern2em \vdots & \ddots & {\mathbf{I}}_{\mathrm{n}}\\ {}{\mathbf{I}}_{\mathrm{n}}\kern0.5em {\mathbf{I}}_{\mathrm{n}}& \cdots & 0\end{array}\right)\ \\ {}\kern0.5em \end{array}\right. $$

6 $$ \mathrm{and}\kern1.25em \mathbf{U}=\left(\begin{array}{c}{\mathbf{U}}_1\\ {}{\mathbf{U}}_2\\ {}.\\ {}.\\ {}.\\ {}{\mathbf{U}}_{\mathrm{M}}\end{array}\right) $$

where N_c_ denotes the putative number of the clusters in each view, **L**_m_ = **S**_m_ - **W**_m_ is the Laplacian matrix of **S**_m_ and **U**_m_ is the assignment matrix of N subjects into N_C_ clusters for view m. The Laplacian matrix reveals the information about the structure of a graph by showing how many edges are linked to each node (subject), and is used to partition the nodes into clusters by leveraging the Laplacian eigenvectors and eigenvalues in spectral clustering method. Spectral clustering is an effective technique for identifying communities of nodes in a graph based on the edges connecting them. This is achieved by dividing the graph nodes into several groups such that nodes in the same group are similar and nodes in different groups are dissimilar to each other (Yu et al. 2019). The first term in the objective function (4) clusters the subjects in each view, while the second term is a constraint to align the clusters in each view. The parameter β is to balance the importance between the network views. Since we treat all views equally, we consider that networks are on the same level and we set β = 1.

To solve the optimization problem (4), we implement the line search algorithm on Stiefel manifold (Yu et al. 2019) to find the optimal solution of the objective function trace(U^T^LU) (Absil et al. 2008). This approach includes three steps. First, we project the negative gradient descent direction of the objective function to the tangent vector space of the Stiefel manifold$\left\{{\mathbf{M}}_{\mathrm{m}}={\mathbf{U}}_{\mathrm{m}}\in {\mathbb{R}}^{\mathrm{N}\times {\mathrm{N}}_{\mathrm{c}}}:{\mathbf{U}}_{\mathrm{m}}^{\mathrm{T}}{\mathbf{U}}_{\mathrm{m}}={\mathbf{I}}_{{\mathrm{N}}_{\mathrm{c}}}\right\},\mathrm{m}=1,2,\dots, \mathrm{M}.$ The gradient descent of the objective function can be defined in a closed form as:

7 $$ -{\nabla}_{\mathrm{U}}\ \mathrm{trace}\left({\mathbf{U}}^{\mathrm{T}}\mathbf{LU}\right)=-\mathbf{LU}={\left({\mathbf{Z}}_1^{\mathrm{T}},{\mathbf{Z}}_2^{\mathrm{T}},\dots, {\mathbf{Z}}_{\mathrm{M}}^{\mathrm{T}}\right)}^{\mathrm{T}} $$

For each manifold **M**_m_, we compute the orthogonal projection to the tangent vector space to get the direction η_m_ which represents also the eigenvector of the Laplacian, then we search for the next point by adding a multiple of this direction to the old iteration point.

8 $$ {\upeta}_{\mathrm{m}}={\mathbf{Z}}_{\mathrm{m}}-\frac{1}{2}{\mathbf{U}}_{\mathrm{m}}\left({\left({\mathbf{U}}_{\mathrm{m}}\right)}^{\mathrm{T}}{\mathbf{U}}_{\mathrm{m}}+{\left({\mathbf{Z}}_{\mathrm{m}}\right)}^{\mathrm{T}}{\mathbf{U}}_{\mathrm{m}}\right) $$

Second, we associate to the new iteration point a retraction to the manifold using single value decomposition and we get the new assignment vector **U**_m_ of the N subjects into N_c_ clusters. We keep updating the line search method until the value of vector **U**_m_ converges to **U**. Finally, we use unsupervised k-means clustering to cluster the elements in **U**. By taking only the k eigenvectors corresponding to the k smallest eigenvalues of **L**_m_, we extract the cluster assignment vector which represents the partition of all subjects in the aligned clusters ${\mathrm{C}}_1^{\mathrm{m}},\dots, {\mathrm{C}}_{{\mathrm{N}}_{\mathrm{c}}}^{\mathrm{m}}$ for each view m.

### Individual-based non-linear fusion of connectional brain network views (step 2)

To estimate the CBT ${\mathbf{A}}_{{\mathrm{n}}_{\mathrm{c}}}^{\mathrm{m}}$ of each cluster n_c_ for the m^*th*^ view, we linearly average all brain networks of subjects belonging to cluster n_c_ in view *m*:

9 $$ {\mathbf{A}}_{{\mathrm{n}}_{\mathrm{c}}}^{\mathrm{m}}=\frac{\sum_{\mathrm{i}\in \mathrm{q}}{\mathbf{V}}_{\mathrm{i}}^{\mathrm{m}}}{\dim \left({\mathrm{n}}_{\mathrm{c}}\right)},\kern1.75em 1\le \mathrm{i}\le \mathrm{N},\kern1.25em 1\le \mathrm{m}\le \mathrm{M},\kern1em 1\le {\mathrm{n}}_{\mathrm{c}}\le {\mathrm{N}}_{\mathrm{c}} $$

where q is the number of subjects in cluster n_c_ for a given view *m*. q can take different values across views and clusters. Next, we merge all ${\mathbf{A}}_{{\mathrm{n}}_{\mathrm{c}}}^{\mathrm{m}}$ across M views using non-linear fusion function ϕ in order to derive an `average’ connectional brain representation of cluster n_c_ across all views:

10 $$ \upphi \left({\left\{{\mathbf{A}}_{{\mathrm{n}}_{\mathrm{c}}}^{\mathrm{m}}\right\}}_{\mathrm{m}=1}^{\mathrm{M}}\right)\mapsto {\mathbf{F}}_{{\mathrm{n}}_{\mathrm{c}}} $$

Ultimately, ϕ non-linearly maps the view-specific CBTs ${\left\{{\mathbf{A}}_{{\mathrm{n}}_{\mathrm{c}}}^{\mathrm{m}}\right\}}_{\mathrm{m}=1}^{\mathrm{M}}$located in different views to a fused brain network ${\mathbf{F}}_{{\mathrm{n}}_{\mathrm{c}}}$ of multi-view networks in cluster n_c_. Thus, we integrate networks sharing the same connectional traits from different manifolds (i.e., views), but within a single cluster. To do so, we leverage the similarity network fusion technique (SNF) proposed by (Wang et al. 2014). SNF enables the fusion of subjects having common neighbors across views so that complementary information can be propagated through the fusion process. Given a cluster n_c_, for each CBT ${\mathbf{A}}_{{\mathrm{n}}_{\mathrm{c}}}^{\mathrm{m}}$ of view *m*,

11 $$ {\mathbf{P}}_{{\mathrm{n}}_{\mathrm{c}}}^{\mathrm{m}}\left(\mathrm{i},\mathrm{j}\right)=\left\{\begin{array}{c}\frac{{\mathbf{A}}_{{\mathrm{n}}_{\mathrm{c}}}^{\mathrm{m}}\left(\mathrm{i},\mathrm{j}\right)}{2{\sum}_{\mathrm{l}\ne \mathrm{i}}{\mathbf{A}}_{{\mathrm{n}}_{\mathrm{c}}}^{\mathrm{m}}\left(\mathrm{i},\mathrm{l}\right)},\kern0.5em \mathrm{j}\ne \mathrm{i}\\ {}\raisebox{1ex}{$1$}\!\left/ \!\raisebox{-1ex}{$2$}\right.,\kern0.5em \mathrm{j}=\mathrm{i}\end{array}\right.\kern0.5em $$

12 $$ {\mathbf{S}}_{{\mathrm{n}}_{\mathrm{c}}}^{\mathrm{m}}\left(\mathrm{i},\mathrm{j}\right)=\left\{\begin{array}{c}\frac{{\mathbf{A}}_{{\mathrm{n}}_{\mathrm{c}}}^{\mathrm{m}}\left(\mathrm{i},\mathrm{j}\right)}{2{\sum}_{\mathrm{l}\in {\mathrm{N}}_{\mathrm{i}}}{\mathbf{A}}_{{\mathrm{n}}_{\mathrm{c}}}^{\mathrm{m}}\left(\mathrm{i},\mathrm{l}\right)},\kern0.5em \mathrm{j}\in {\mathrm{N}}_{\mathrm{i}}\\ {}0,\kern0.5em \mathrm{otherwise}\end{array}\right. $$

Note that **P** carries all information about ROIs similarities of each subject to all other ROIs, whereas **S** only encodes the similarity to the **K**_***n***_ most similar ROIs. N_i_ denotes the set of most q closed ROIs (neighbors) to the target ROI R_i_. To find the set N_i_, we use the K-nearest neighbors (KNN) algorithm. Next, we compute iteratively the status matrices ${\mathbf{P}}_{{\mathrm{n}}_{\mathrm{c}}}^{\mathrm{m}}$ by using the following equation (Wang et al. 2014):

13 $$ {\mathbf{P}}_{{\mathrm{n}}_{\mathrm{c}}}^{\mathrm{m}}={\mathbf{S}}_{{\mathrm{n}}_{\mathrm{c}}}^{\mathrm{m}}\times \left(\frac{\sum \limits_{\mathrm{t}\ne \mathrm{m}}{\mathbf{P}}_{{\mathrm{n}}_{\mathrm{c}}}^{\mathrm{t}}}{\mathrm{M}-1}\right)\times {\left({\mathbf{S}}_{{\mathrm{n}}_{\mathrm{c}}}^{\mathrm{m}}\right)}^{\mathrm{T}},\mathrm{m}\in \left\{1,\dots, \mathrm{M}\right\} $$

For each cluster n_c_ and view m, we update the similarity matrix ${\mathbf{P}}_{{\mathrm{n}}_{\mathrm{c}}}^{\mathrm{m}}$ by diffusing the global structure of other networks $\frac{\sum_{\mathrm{t}\ne \mathrm{m}}{\mathbf{P}}_{\mathrm{m}}^{\mathrm{t}}}{\mathrm{m}-1}$ along the sparse structure ${\mathbf{S}}_{{\mathrm{n}}_{\mathrm{c}}}^{\mathrm{m}}$ of the current view m. After N_t_ iterations, we compute the average of the diffused matrices ${\mathbf{P}}_{{\mathrm{n}}_{\mathrm{c}}}^{\mathrm{m}}$across the different M views and we get the fused CBT representing the cluster n_c_ across views using the following equation:

14 $$ {\mathbf{F}}_{{\mathrm{n}}_{\mathrm{c}}}=\frac{\sum_{\mathrm{m}=1}^{\mathrm{M}}{\mathbf{P}}_{{\mathrm{n}}_{\mathrm{c}}}^{\mathrm{m}}}{\mathrm{M}} $$

### Linear fusion (step 3)

After obtaining the cluster-based CBTs ${\left\{{\mathrm{F}}_{{\mathrm{n}}_{\mathrm{c}}}\right\}}_{{\mathrm{n}}_{\mathrm{c}}=1}^{{\mathrm{N}}_{\mathrm{c}}}$, we linearly average them into a single final CBT denoted as **C**:

15 $$ \mathbf{C}=\frac{\sum_{{\mathrm{n}}_{\mathrm{c}}=1}^{{\mathrm{N}}_{\mathrm{c}}}{\mathbf{F}}_{{\mathrm{n}}_{\mathrm{c}}}}{{\mathrm{N}}_{\mathrm{c}}} $$

MVCF-Net Algorithm 1: *Joint multi-View Network Clustering and Fusion*.
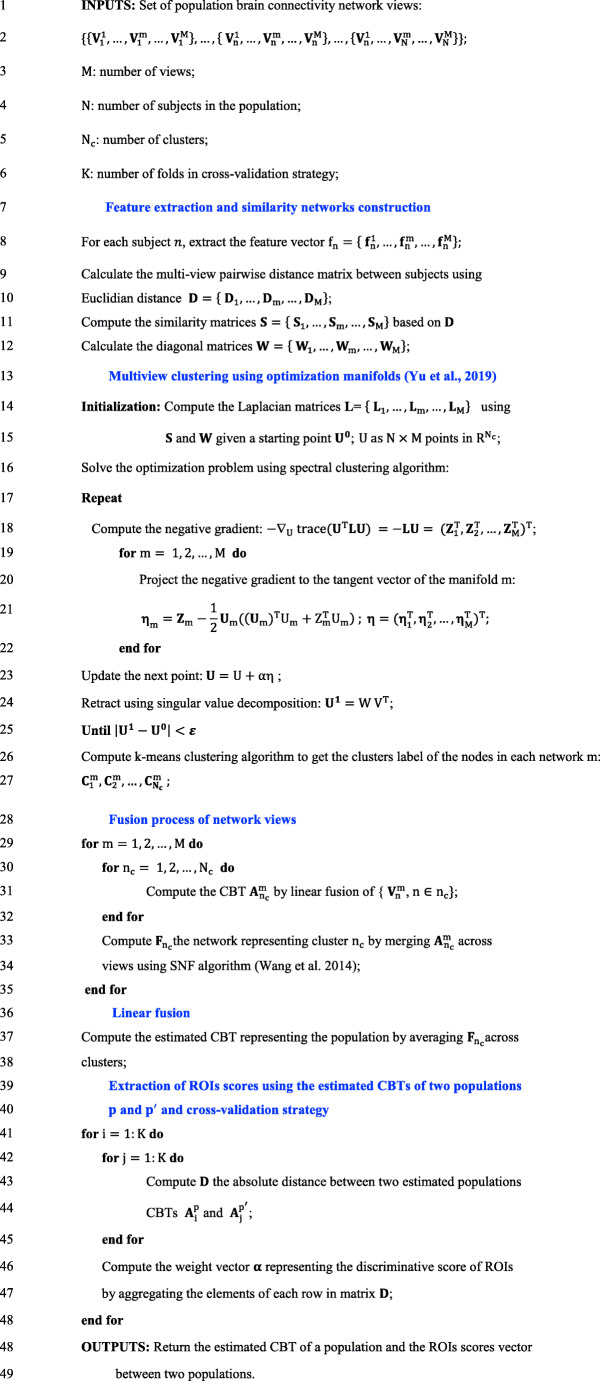


### Evaluation strategy of connectional brain template representativeness

We evaluate both the centeredness and representativeness of the estimated CBT for a given population using two evaluation metrics: (i) the mean Frobenius distance as well as (ii) the Pearson correlation between the estimated CBT and the brain networks of all subjects across views in the given population. For each view m, we compute the mean Frobenius distance ${\mathrm{d}}_{\mathrm{F}}^{\mathrm{m}}$ between the estimated CBT and all brain networks, then we calculate the average of ${\mathrm{d}}_{\mathrm{F}}^{\mathrm{m}}$ across the views. Likewise, we compute the mean Pearson correlation r^m^ for each view between the predicted CBT and all brain networks belonging to a given population, then we linearly average r^m^ across views. The Frobenius distance and the Pearson correlation between two matrices **G** = (g_ij_) and **H** = (h_ij_) where 1 ≤ i, j ≤ N are calculated as follow:

16 $$ {\mathrm{d}}_{\mathrm{F}}\left(\mathbf{G},\mathbf{H}\right)=\sqrt{\sum_{\mathrm{i}}{\sum}_{\mathrm{j}}{\left|{\mathrm{g}}_{\mathrm{i}\mathrm{j}}-{\mathrm{h}}_{\mathrm{i}\mathrm{j}}\right|}^2} $$

17 $$ \mathrm{r}\left(\mathbf{G},\mathbf{H}\right)=\frac{\sum_{\mathrm{i}}{\sum}_{\mathrm{j}}\left({\mathrm{g}}_{\mathrm{i}\mathrm{j}}-\overline{\mathrm{g}}\right)\left({\mathrm{h}}_{\mathrm{i}\mathrm{j}}-\overline{\mathrm{h}}\right)}{\sqrt{\left({\sum}_{\mathrm{i}}{\sum}_{\mathrm{j}}{\left|{\mathrm{g}}_{\mathrm{i}\mathrm{j}}-\overline{\mathrm{g}}\right|}^2\right)\left({\sum}_{\mathrm{i}}{\sum}_{\mathrm{j}}{\left|{\mathrm{h}}_{\mathrm{i}\mathrm{j}}-\overline{\mathrm{h}}\right|}^2\right)}} $$

where $\overline{\mathrm{g}}= mean\ \left(\boldsymbol{G}\right)$and $\overline{\mathrm{h}}= mean\ \left(\boldsymbol{H}\right)$. For evaluating the reproducibility of the estimated CBTs, we use K-fold cross-validation for validating and testing. We randomly split each group in the given population of multi-view brain networks into K sub-populations. For each sub-population, we generate a CBT and we measure its Frobenius distance to views. For better visualization of the results and for easy comparison between methods, we further normalize the Frobenius distances for each fold using the following formula:

18 $$ {\mathrm{d}}_{\mathrm{F}}^{\prime }=\left({\mathrm{d}}_{\mathrm{F}}-{\mathrm{mean}}_{\mathrm{i}}\right)/\left({\max}_{\mathrm{i}}-{\mathrm{mean}}_{\mathrm{i}}\right)+1.5 $$

where mean_i_ and max_i_ denote respectively the average and the maximum Frobenius distances in fold *i*.

### Evaluation strategy of connectional brain template discriminability

**Fig. 3:**
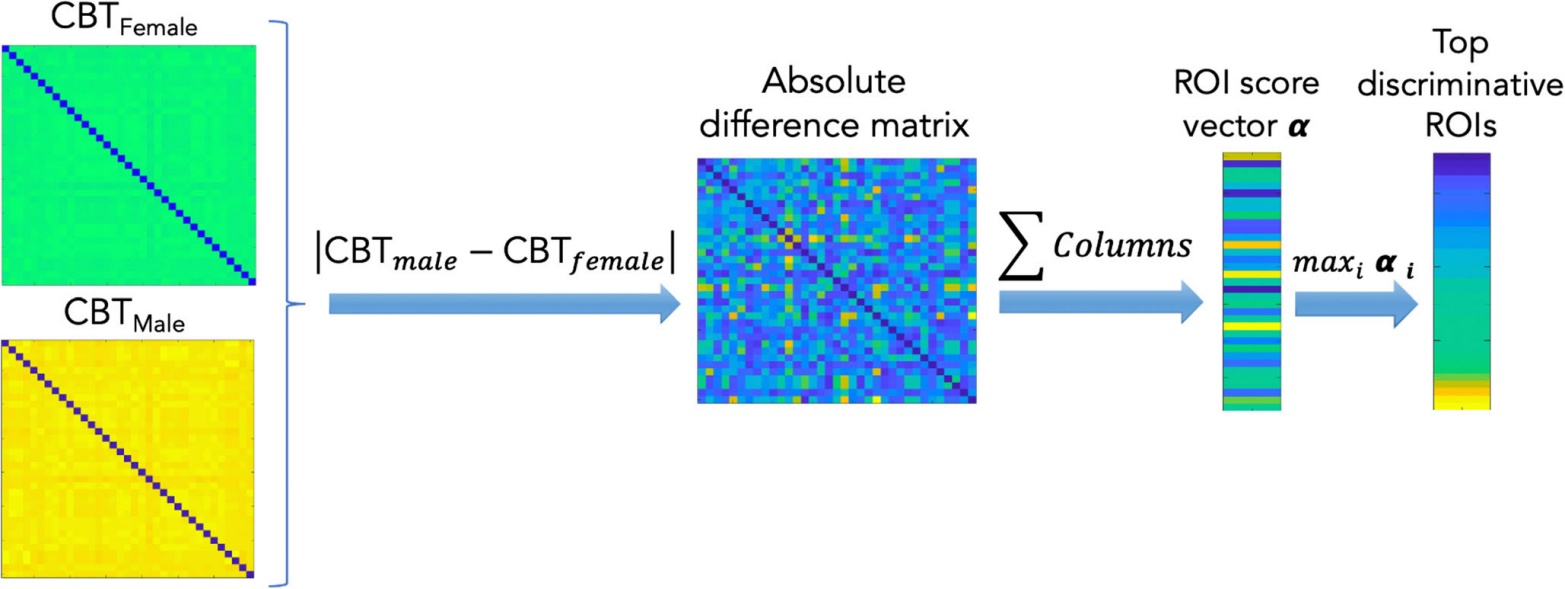
*Identification of regions of interest (ROIs) scores using MVCF-Net method.* First, we calculate the absolute difference between two estimated connectional brain templates (CBTs) to generate the absolute difference matrix. Secondly, we aggregate the column elements of each row in the absolute difference matrix to produce a score vector assigning the weight for each ROI. Finally, we decreasingly rank the elements of score vector to get the top discriminative ROIs

In this part, we aim to test the discriminability of the estimated CBTs by identifying the top brain ROIs that distinguish between two groups. This experiment evaluates the performance of a given method in relation with the discriminability of the ROIs. To do so, we estimate a CBT for each group, then by computing the difference between both templates, we identify the top ROIs distinguishing between both groups. Next, we compute the overlap (in %) between the top discriminative ROIs found by MVCF-Net and a supervised machine learning method based on multiple kernel learning (MKL) (Fig. 3). Both methods are detailed below.

### Identification of top discriminative ROIs using the estimated CBTs

To assess the reproducibility of our proposed method, we use K-fold cross validation strategy to partition samples in each population (male/female) into K groups (folds). We denote by *p*_*i*_ the fold i of group 1 (e.g., male) and ${p}_j^{\prime }$the fold j of group 2, where 1 ≤ i, j ≤ K. After computing the estimated CBTs of all folds for both populations, we compute the average absolute difference between all possible pair combinations of estimated CBTs. Each combination includes CBTs from both fold groups *p* and *p*′, then we define an N_r_ × N_r_ matrix **T** representing the cumulative absolute differences between all pairs of CBTs:

19 $$ \mathbf{T}={\sum}_{\mathrm{i},\mathrm{j}=1}^{\mathrm{K}}\mid {\mathbf{A}}_{\mathrm{i}}^{\mathrm{p}}-{\mathbf{A}}_{\mathrm{i}\mathrm{j}}^{{\mathrm{p}}^{\prime }}\mid, \kern1.5em 1\le \mathrm{i},\mathrm{j}\le \mathrm{K} $$

where ${\mathrm{A}}_{\mathrm{i}}^{\mathrm{p}}$ denotes the CBT of group 1 from fold i and ${\mathbf{A}}_{\mathrm{j}}^{\mathrm{p}\prime }$ is CBT of group 2 from fold j. By aggregating the elements of each row in **T**, we get the weight score α_i_ assigned to the ROI R_i_. The obtained α_i_ denotes the cumulative Euclidian distance from R_i_ to all other ROIs R_j_ (j ≠ i). Next, we rank the elements in score vector **α** decreasingly to identify the top discriminative ROIs having the highest scores. The pipeline steps of top discriminative ROIs are illustrated in Fig. 3. 훼_푖_ is calculated as follows:

### Reproducibility of top discriminative ROIs

Next, we aim to evaluate the reproducibility of the top discriminative ROIs revealed by two CBTs, each derived from a particular population. To this aim, we propose to use an independent machine-learning methodology for supervised feature selection, namely multiple kernel learning (MKL), and compare the ROIs identified by MVCF-Net and MKL. MKL is a technique that learns an optimal combined kernel from predefined basic kernels (e.g. information coming from multiple sources by maximizing separability between them). Specifically, MKL was shown to be powerful in classification task that distinguishes between classes while identifying the most discriminative features between them (Varma and Babu 2009). Given a labeled sample with its corresponding feature vector, we train an SVM classifier that learns a weight score for each feature measuring its discriminative power in the target classification task. For each network view *m*, we use a K-fold randomized partition to divide the data into *K* subpopulations. Let *p* denote population 1 and *p*′ population 2. For each combination of subpopulations *p*_*i*_ and ${p}_j^{\prime }$, where 1 ≤ i, j ≤ K, we construct a feature vector ${\mathbf{F}}_{\mathrm{n}}^{\mathrm{m}}$ for each subject *n* in both subpopulations *p* and *p*′ using the vectorized upper triangular part of the connectivity matrix ${\mathbf{V}}_{\mathrm{n}}^{\mathrm{m}}$, and we assign its label ${\mathbf{y}}_{\mathrm{n}}^{\mathrm{m}}$ ∈ {±1} indicating the population class. Using ${\mathbf{f}}_{\mathrm{n}}^{\mathrm{m}},{\mathbf{y}}_{\mathrm{n}}^{\mathrm{m}}$ and the subpopulations *p*_*i*_ and ${p}_j^{\prime }$as inputs to train the SVM classifier, we use a wrapper method to estimate a weight vector${\mathbf{x}}_{\mathrm{i},\mathrm{j}}^{\mathrm{m}}$ which assigns a learned weight quantifying the importance of each feature (i.e., brain connectivity) in distinguishing between two classes. We compute the total weight vector **x** by cumulating ${\mathbf{x}}_{\mathrm{i},\mathrm{j}}^{\mathrm{m}}$ across all views and all combinations of subpopulations:

20 $$ \mathbf{x}=\sum \limits_{\mathrm{i},\mathrm{j}=1}^{\mathrm{k}}\sum \limits_{\mathrm{m}=1}^{\mathrm{M}}{\mathbf{x}}_{\mathrm{i},\mathrm{j}}^{\mathrm{m}} $$

We apply anti-linearization to transform the weight vector **x** into a square matrix **B** where each element **B**(i, j) represents the learned weight assigned to brain connections between ROIs R_i_ and R_j_. Next, by summing up the weights of all connections involving R_i_ to other ROIs, we obtain the weight score α_i_ that quantifies the discriminative power of R_i_. α_i_ is then calculated as follows:

21 $$ {\upalpha}_{\mathrm{i}}=\sum \limits_{\mathrm{j}\ne \mathrm{i}}\mathbf{B}\left(\mathrm{i},\mathrm{j}\right),1\le \mathrm{j}\le {\mathrm{N}}_{\mathbf{r}} $$

**Fig. 4:**
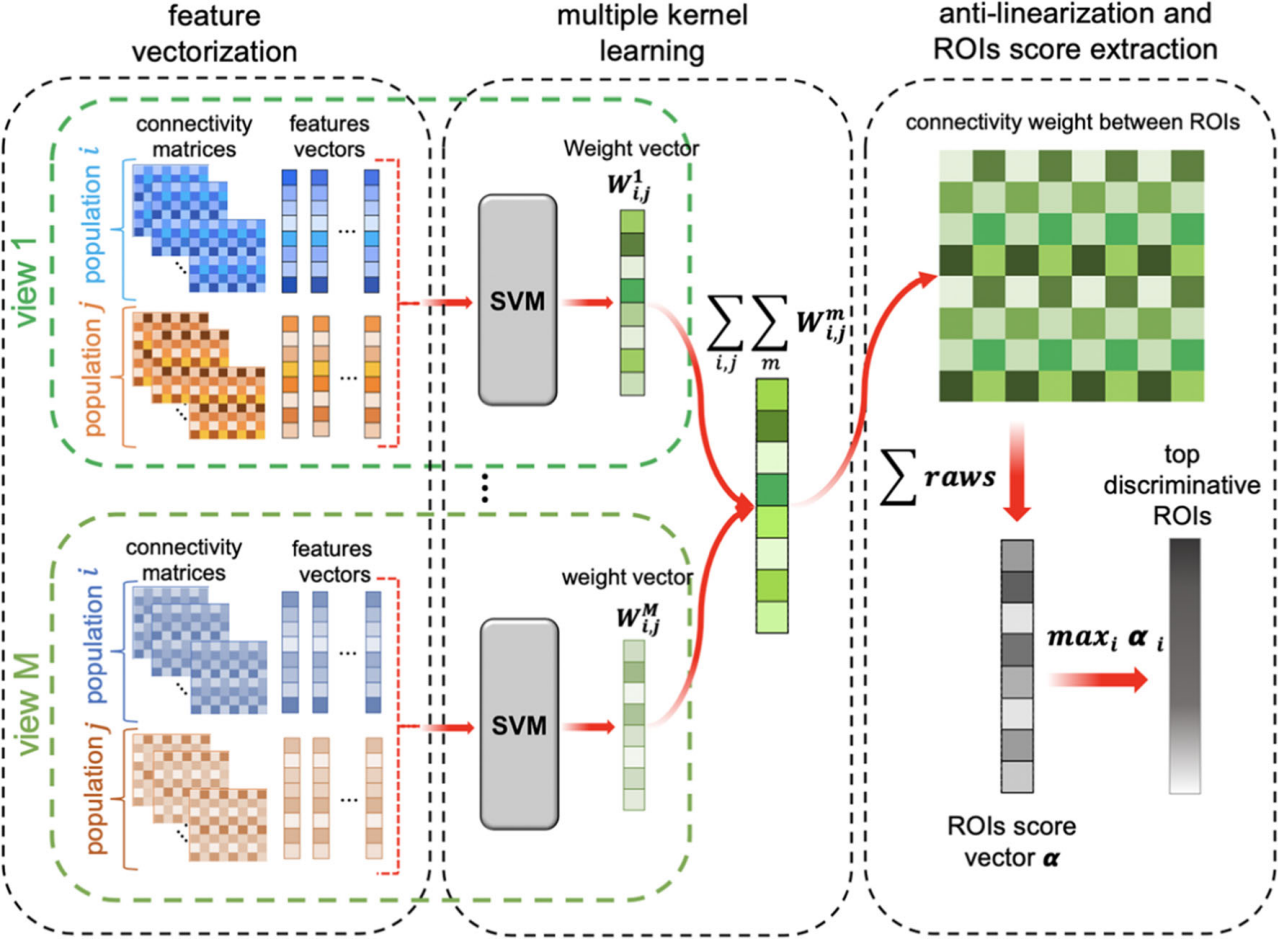
*Identification of the top discriminative ROIs using multiple kernel learning (MKL)*. First, we linearize the multi-view brain connection networks for training and testing brain networks through the vectorization of the upper triangular part of each population matrices to generate a feature vector for each brain network. Second, for each view *m*, we apply MKL based on support vector machine (SVM) to obtain a weight vector **x**^m^ quantifying the discriminability of each brain feature (i.e., brain connectivity between two anatomical regions of interest (ROIs)). Next, by summing the weight vectors **x**^m^ across views, we obtain the total weight vector **x** for a particular ROI. We then use anti-linearization to transform the weight vector into a matrix where each element represents the connectivity weight between two ROIs. Specifically, anti-linearization is the inverse of features vectorization where the weight vector represents the upper triangular part of the resulting symmetrical connectivity matrix. By aggregating the columns of the resulting matrix, we obtain the score vector denoting the discriminative power of each ROI. Finally, we rank brain ROIs according to their highest scores

Finally, we select the top discriminative ROIs using the highest scores α_**i**_, where 1 ≤ i ≤ N_**r**_ . The identification pipeline of top discriminative ROIs using MKL technique is illustrated in Fig. 4.

## Results

### Comparison methods

In this work, we propose a robust multi-view clustering and fusion network MVCF-Net method for CBT estimation which can simultaneously capture shared and distinct traits of a population lying on different views and identify the top discriminative ROIs marking gender differences. For comparative evaluation, we benchmark MVCF-Net against a state-of-the-art method (SCA) introduced in (Dhifallah et al. 2019).

### Parameter setting

**Fig. 5:**
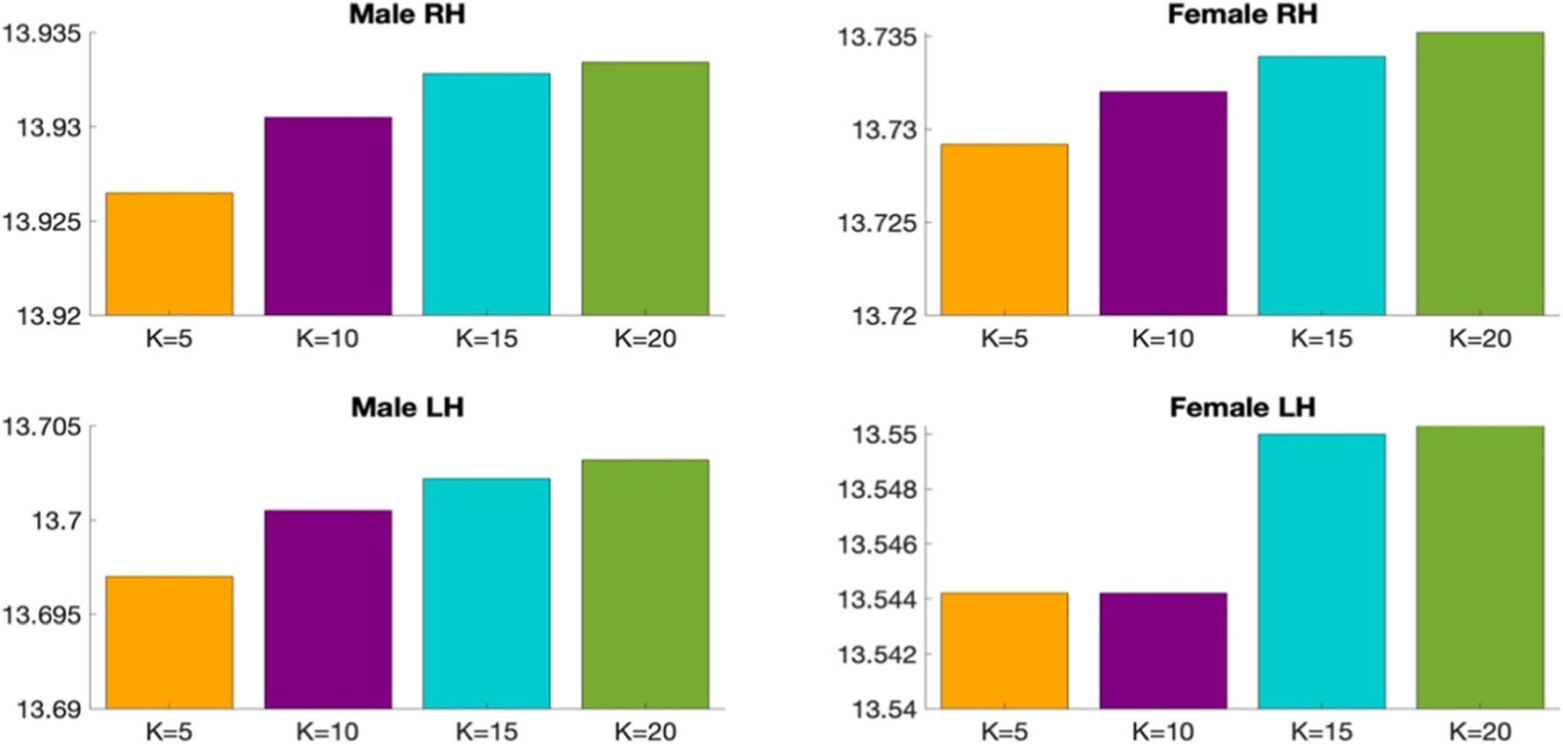
Average Frobenius distance between the estimated CBT by MVCF-Net and all CMNs in the left (LH) and right (RH) hemispheres as we vary the number of selected neighbors for KNN

**Fig. 6:**
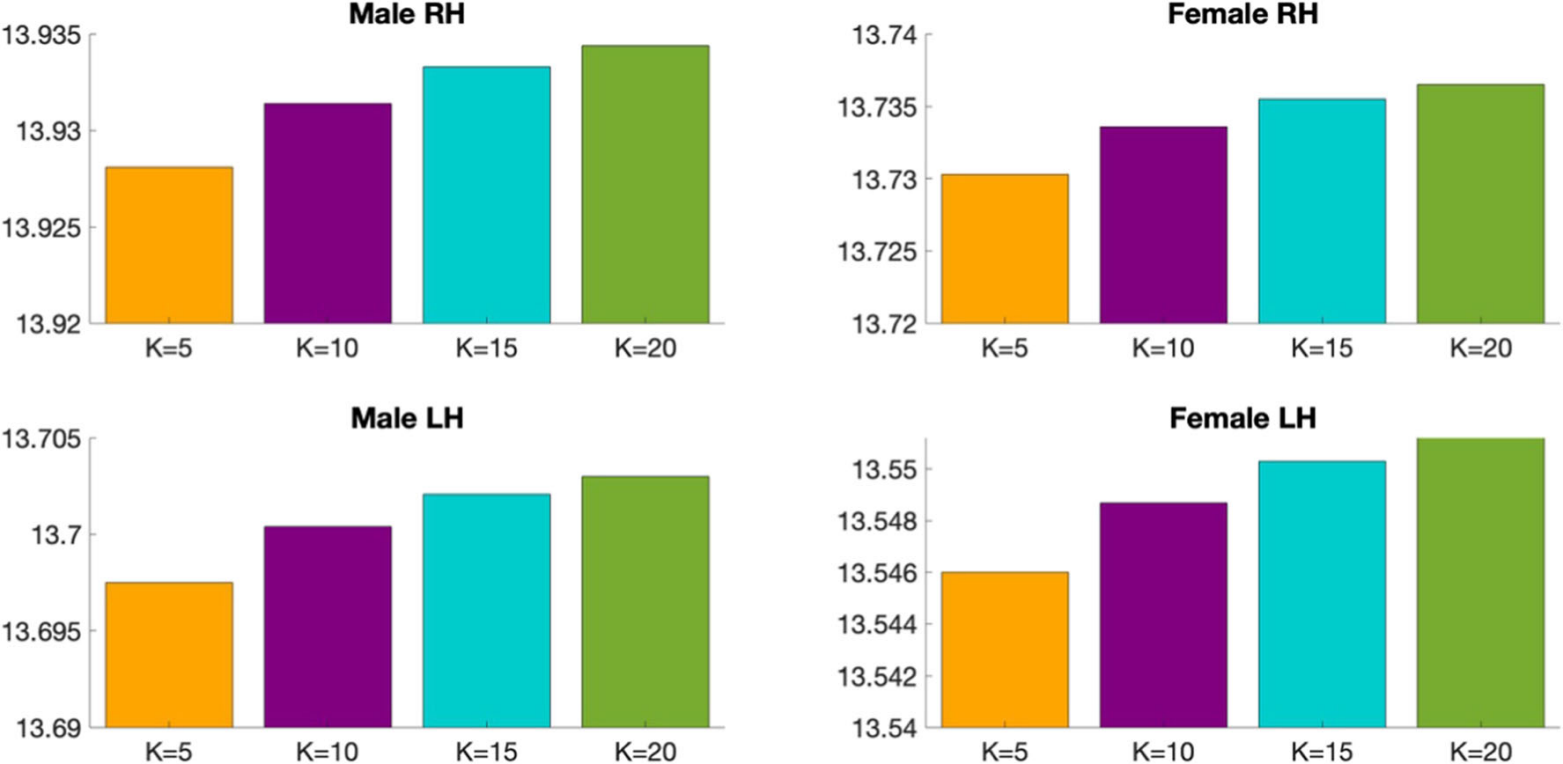
Average Frobenius distance between the estimated CBT by SCA (Dhifallah et al. 2019) and all CMNs in the left (LH) and right (RH) hemispheres as we vary the number of selected neighbors for KNN

**Fig. 7:**
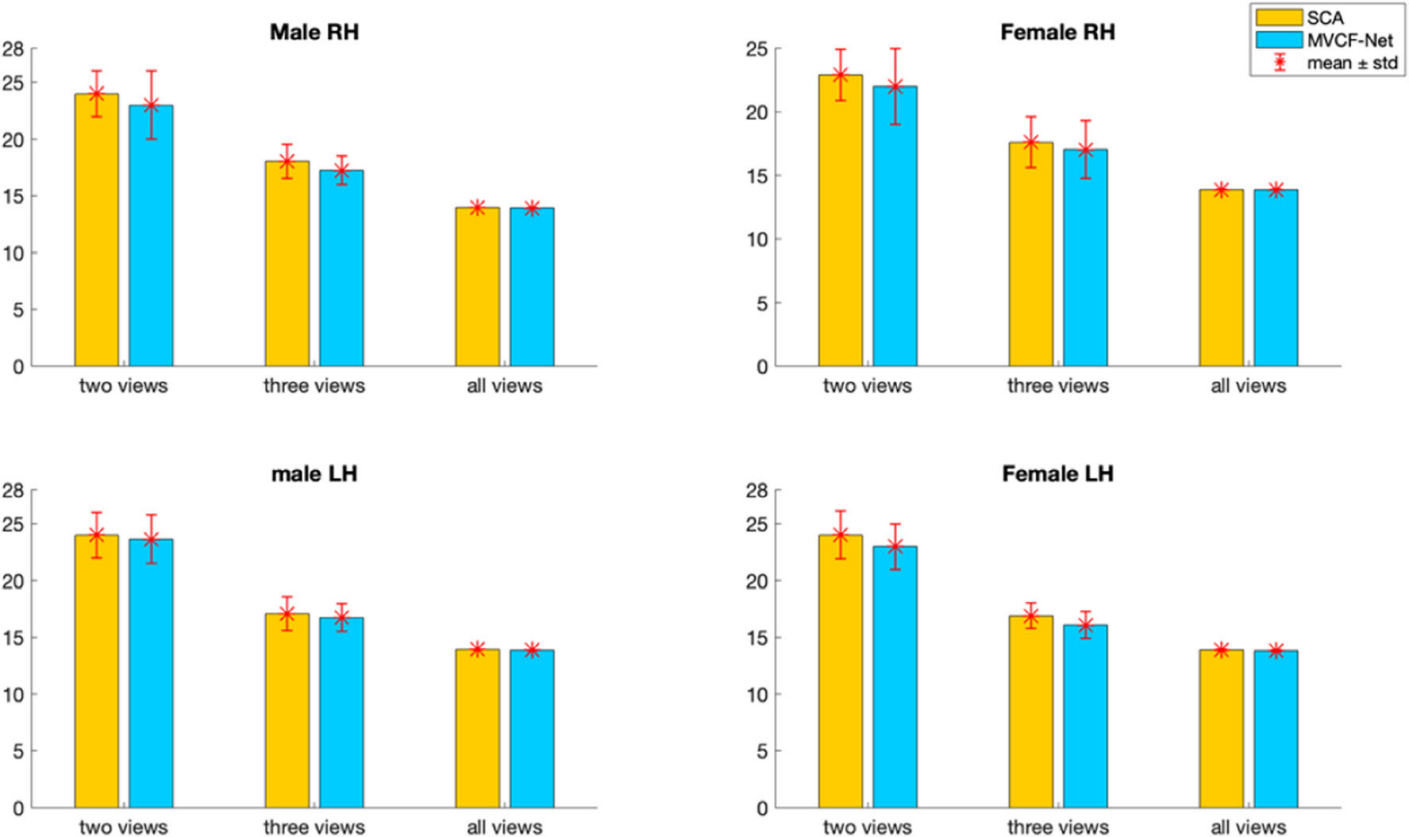
Average Frobenius distance between the estimated morphological CBT and all CMNs in the left (LH) and right (RH) hemispheres using our method MVCF-Net in comparison with SCA (Dhifallah et al. 2019) as we vary the number of selected views constructing the CMNs from 2 to 4 views. Each bar represents the average Frobenius distance and its standard deviation of all possible combinations for a given number of views

**Fig. 8:**
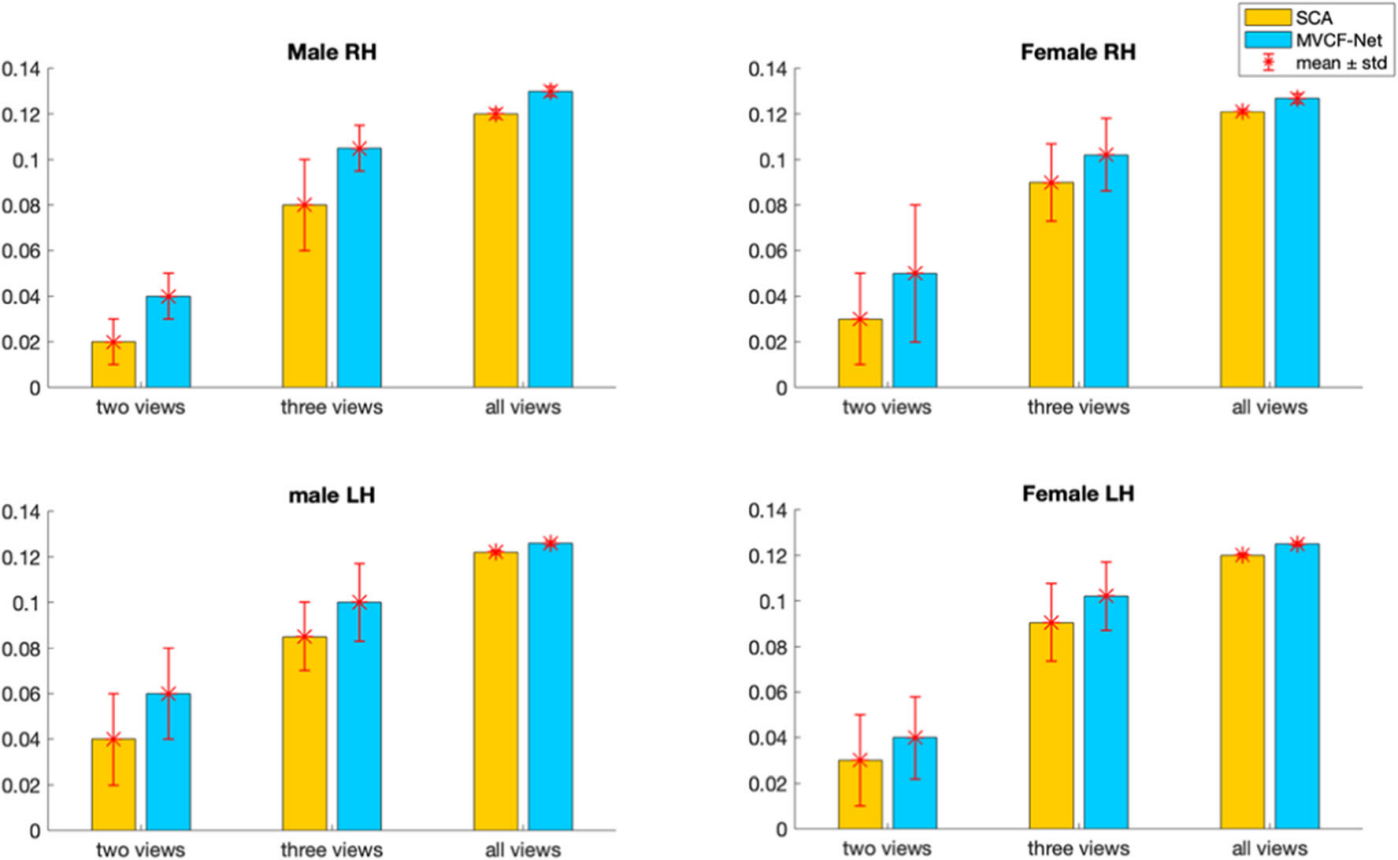
Average Pearson correlation between the estimated morphological CBT and all CMNs in the left (LH) and right (RH) hemispheres using our method MVCF-Net in comparison with SCA (Dhifallah et al. 2019) as we vary the number of selected views constructing the CMNs from 2 to 4 views. Each bar represents the average Pearson correlation and its standard deviation of all possible combinations for a given number of views

We list below the parameters used in our methodology and comparison methods: (1) K_*n*_: the number of selected neighbors for KNN (2) N_*c*_: the number of clusters for k-means clustering and (3) *M*: the number of views:

- Number of clusters N_c_. In fact, we use a grid search strategy that considers all parameter combinations by varying the number of clusters N_*c*_ in the range [2,15] in order to determine the best N_*c*_ that achieves the minimum Frobenius distance and the maximum Pearson correlation for the multi-view brain networks across all methods (ours and SCA). We found that the optimal number of clusters *N*_*c*_ is equal to 3 across all methods.
- K_n_ in KNN algorithm. We also investigate the best number of *K*_*n*_ nearest neighbors used in KNN method. We vary *K*_*n*_ in the set (5,10,15,20) and we find that *K*_*n*_ = 5 achieves the minimum Frobenius distance between the estimated templates and all population networks for each method, independently. Figures 5 and 6 display the average Frobenius distance between the estimated CBT and all CMNs using our method and SCA (Dhifallah et al. 2019) while varying the number of *K*_*n*_ nearest neighbors. Noticeably, setting K_*n*_ = 5 achieves the best results across all methods. We also use the grid search strategy to identify the best combination of the parameters N_c_ and K_n_ dependently.
- Number of views M. We vary the number of selected views to build the subject-specific CMNs from 2 to 4 views. For each selected number of views, we assess all possible combinations of views out of the existing 4 views (e.g., we have ${\mathrm{C}}_4^2$ possible combinations of M = 2 out of 4 views). We report the average Frobenius distance and the average Pearson correlation between the estimated morphological CBT and all CMN views in the left (LH) and right (RH) hemispheres using our method MVCF-Net in comparison with SCA (Dhifallah et al. 2019) respectively represented in **Figs**. 7 and 8.

Figures 7 and 8 display the average Frobenius distance and the average Pearson correlation between the estimated morphological CBT and all CMNs using our method MVCF-Net in comparison with SCA (Dhifallah et al. 2019) as we vary the number of selected views constructing the subject-specific CMNs. For each selected number of views (e.g. 2 views, 3 views, all views), we compute the average of metric (e.g. Frobenius distance, Pearson correlation) using all combination of brain networks. Noticeably, including all views together (e.g. four cortical attributes) achieves the best results for the average Frobenius distance and the average Pearson correlation in the left (LH) and right (RH) hemispheres across all methods (Ours and SCA).

### CBT representativeness and centeredness

**Fig. 9:**
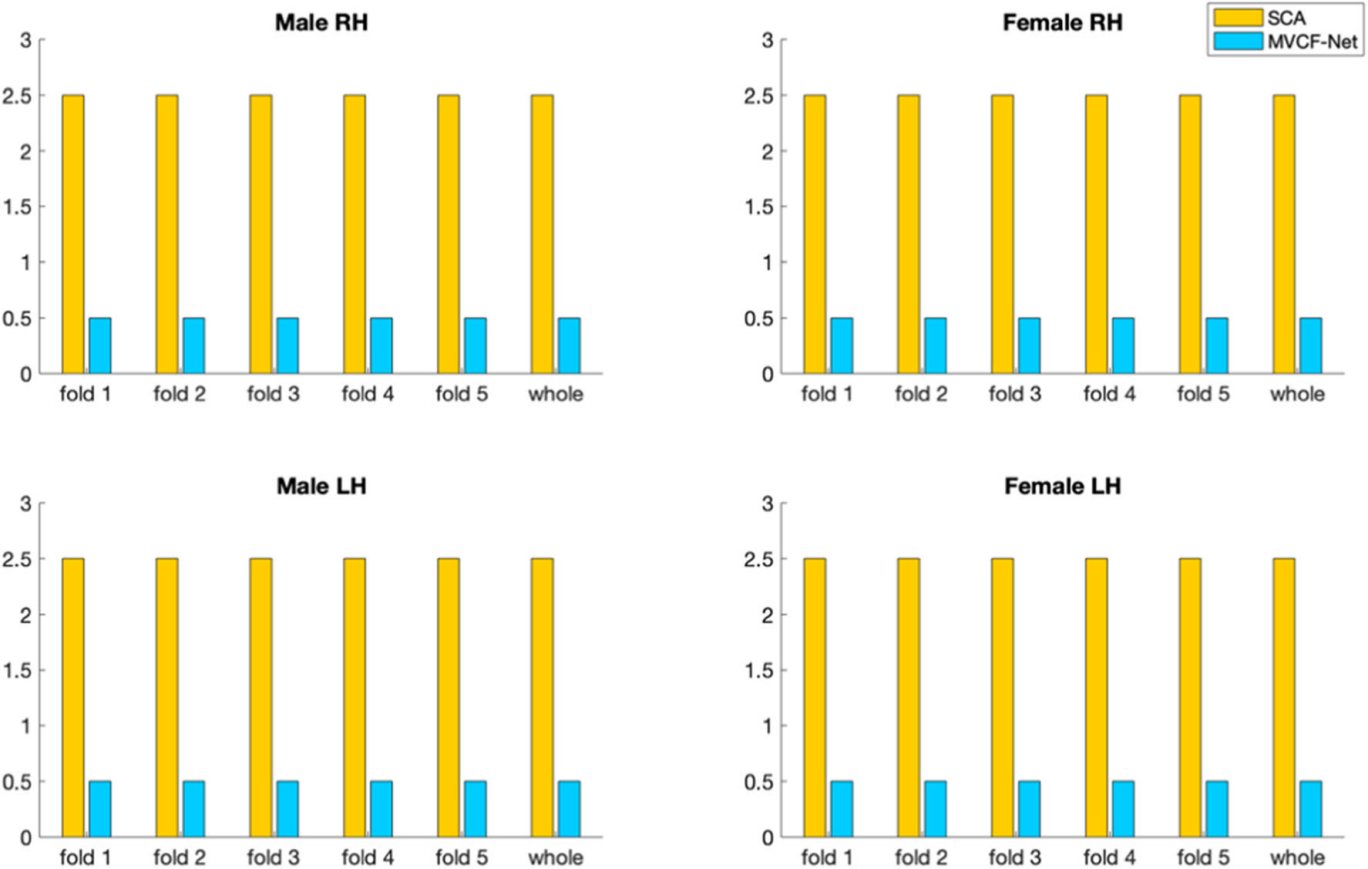
Evaluation of the normalized Frobenius distance between the estimated morphological CBT and all multi-view brain networks for male and female populations in left and right hemispheres (LH and RH) using our method MVCF-Net in comparison with SCA (Dhifallah et al. 2019)

**Fig. 10:**
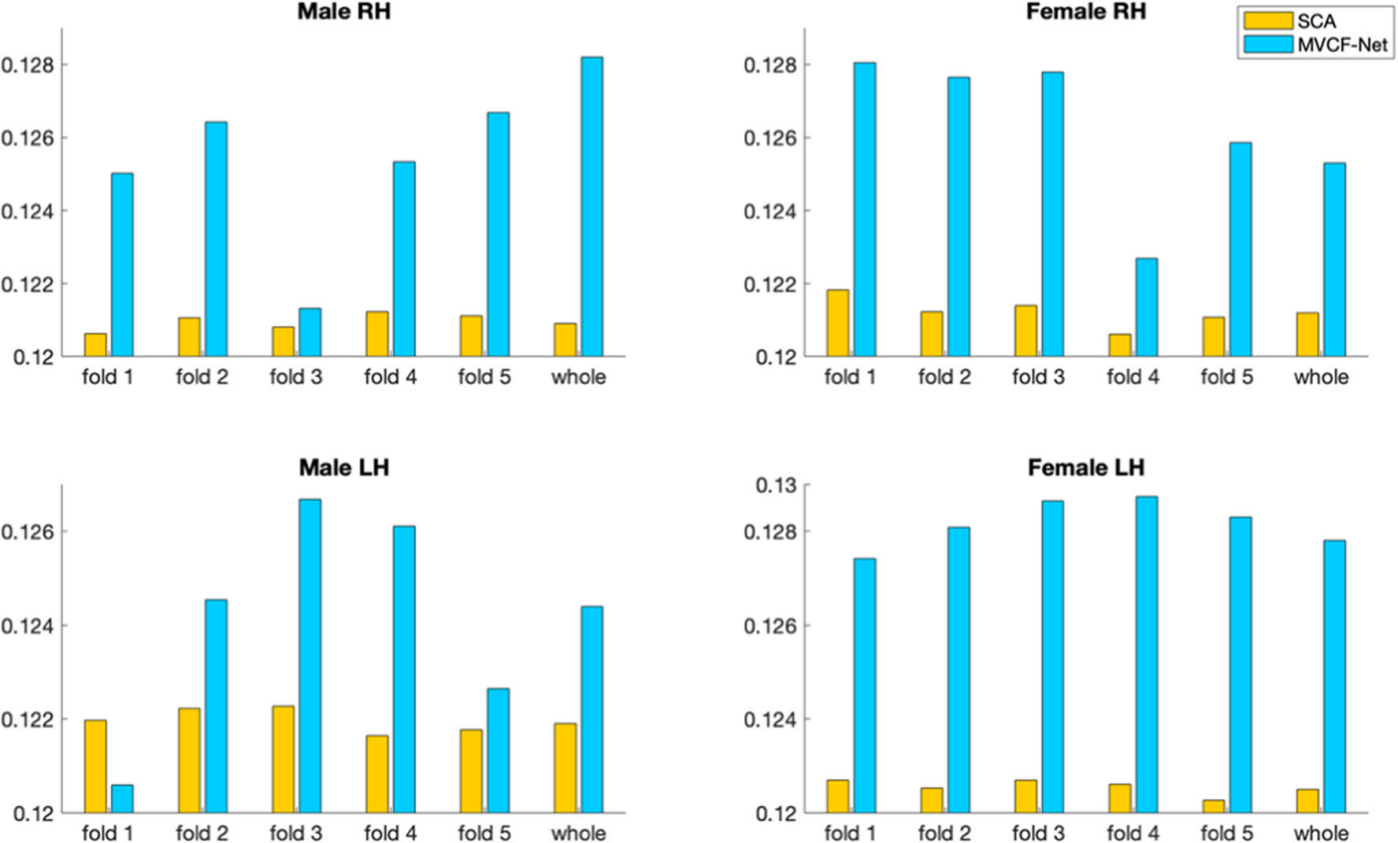
Evaluation of Pearson correlation between the estimated morphological CBT and all multi-view brain networks for male and female populations in left and right hemispheres (LH and RH) using our method MVCF-Net in comparison with SCA (Dhifallah et al. 2019)

We evaluate the representativeness of the proposed CBT by computing the mean Frobenius distance and the Pearson correlation between the estimated brain network and all different views (4 views) in each population for SCA as well as for MVCF-Net in left and right hemispheres. To better visualize the difference in performance between MVCF-Net and SCA, we plot the normalized Frobenius distance in Fig. 9. Also, we randomly partition our data into 5 folds to evaluate the reproducibility of our results across folds as well as when using the whole dataset. As illustrated in Figs. 9 and 10, our MVCF-Net provides the best centered CBTs for male and female populations in both hemispheres. Based on both evaluation metrics, MVCF-Net method outperforms SCA by achieving the minimum Frobenius distance and the maximum correlation between the estimated CBT and all views for whole and sub-populations (5 folds) in each hemisphere. Excluding one male LH sub-population, MVCF-Net achieves the maximum correlation comparing to SCA. A smaller Frobenius distance indicates a more centered CBT with respect to all individuals in the population and all views. Clearly, MVCF-Net estimates the most centered brain template for each population. Further, our method stands out in performance in comparison with SCA as we vary the number of selected views constructing the morphological CMNs from 2 to 4 views. As illustrated in Figs. 7 and 8, our MVCF-Net provides the best centered CBTs for male and female populations by achieving the optimal averages in both Frobenius distance and Pearson correlation between the estimated morphological CBT and all CMNs in the left (LH) and right (RH) hemispheres when the number of views is equal to 2, 3 and 4, respectively.

We note that MVCF-Net significantly (*p* value <0.001) outperforms SCA comparison method in terms of centeredness across all populations in both hemispheres based on two-tailed paired t-test.

### CBT discriminability

In addition to being well-centered, we demonstrate that MVCF-Net generates a well-discriminative CBT able to easily spot gender-distinctive brain regions. In particular, we identify the top 15 discriminative ROIs distinguishing between male and female populations for both hemispheres using the estimated CBTs representing each group. To compare the performance between MVCF-Net and SCA methods, we evaluate the reproducibility of the top 15 discriminative ROIs distinguishing between gender populations in comparison with a feature selection method, namely MKL. Next, we compute the overlap between the most discriminative ROIs identified using our method and those using MKL.

Table 3 displays the overlap in % between the top 15 discriminative ROIs identified using (i) MKL and (ii) the absolute difference between the two estimated CBTs by MVCF-Net and SCA, respectively. We demonstrate that our method achieves an overlap percentage of 60% in identifying the most discriminative brain regions in the left hemisphere between genders and 46.67% in the right hemisphere. While SCA method reaches only an overlap percentage of 53.33% and 33.33% in the left hemisphere and the right hemisphere, respectively. Table 4 displays the overlap in % between the top 20 discriminative ROIs identified using MKL and the absolute difference between the two estimated CBTs. Specifically, our method achieves an overlap percentage of 65% in identifying the most discriminative brain regions in the left hemisphere between genders and 60% in the right hemisphere, while SCA reaches only an overlap rate of 45% for left hemisphere and 55% for the right hemisphere. We notice that the overlap rates between the most discriminative ROIs identified using (i) MKL and MVCF-Net methods as well as using (ii) MKL and SCA methods are higher in the left hemisphere compared to the right hemisphere. Our finding supports the evidence that strong gender-related differences are more prevalent in the left hemisphere (Tian et al. 2011).

**Table 3:** Matching rate in % between the top 15 discriminative ROIs distinguishing between male and female populations identified by (i) MKL and (ii) the difference between the estimated CBTs by SCA and our method for the right and left hemispheres (RH and LH)

Datasets	Male / Female	
	LH	RH
SCA	53.33%	33.33%
Ours	60%	46.67%

**Table 4:** Matching rate in % between the top 20 discriminative ROIs distinguishing between male and female populations identified by (i) MKL and (ii) the difference between the estimated CBTs by SCA and our method for the right and left hemispheres (RH and LH)

Datasets	Male / Female	
	LH	RH
SCA	60%	45%
Ours	65%	55%

**Fig. 11:**
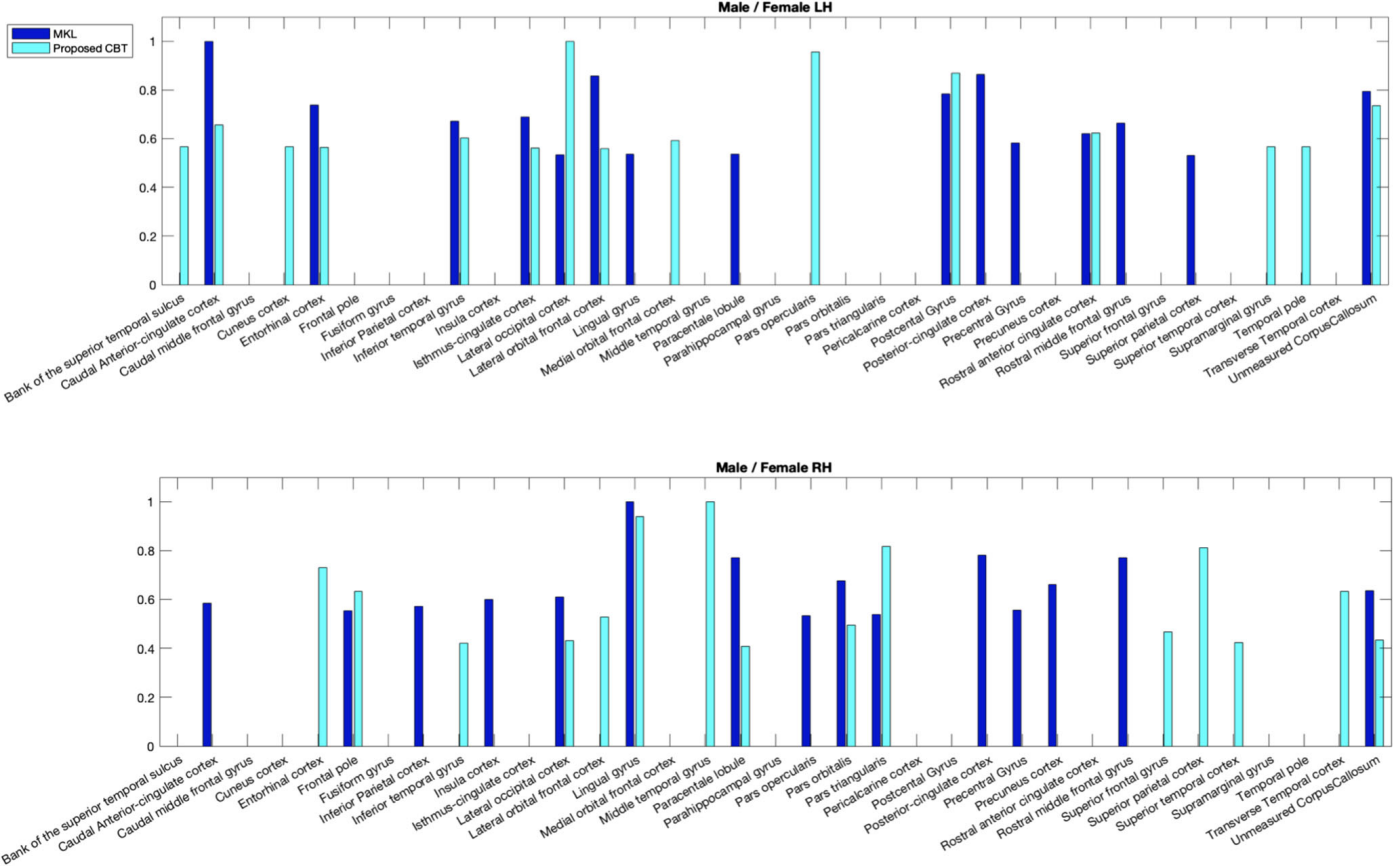
*Evaluating the discriminability of the estimated population-specific connectional brain template by MVCF-Net.* We identify the top 15 discriminative ROIs using multiple-kernel learning (MKL) and the absolute difference between male and female CBTs in the right and left hemispheres (RH and LH). For each of top identified 15 ROIs, we display their discriminative weight

In Fig. 11, we visualize the top 15 discriminative ROIs that distinguish between gender populations for left and right hemispheres using MKL and MVCF-Net, respectively. We plot the discriminability weight of ROIs using the normalized score vector 휶. We note the most two discriminative ROIs selected by MVCF-Net differentiating between male and female populations include the lateral occipital cortex (region 12) followed by the pars opercularis (region 19) for the left hemisphere. These regions are correlated with processing of visuospatial and motion information (Amunts et al. 2007). For the right hemisphere, the two highly ranked discriminative ROIs identified by our method included the middle temporal gyrus (region 16) and lingual gyrus (region14) which are correlated with brain size, gray-matter volume and concentration (Takahashi et al. 2011; Yang et al. 2017). Table 5 displays the top 5 discriminative ROIs distinguishing between gender populations using MVCF-Net for both RH and LH. These regions are consistent with the literature findings investigating the gender fingerprint, where they were shown to be involved in visuospatial processing, cognitive performance, emotion and facial expression. Precisely, the most discriminative regions selected by our method explain the difference in integration, communication, reaction and memories abilities between human genders (Diano et al. 2017).

**Table 5:**
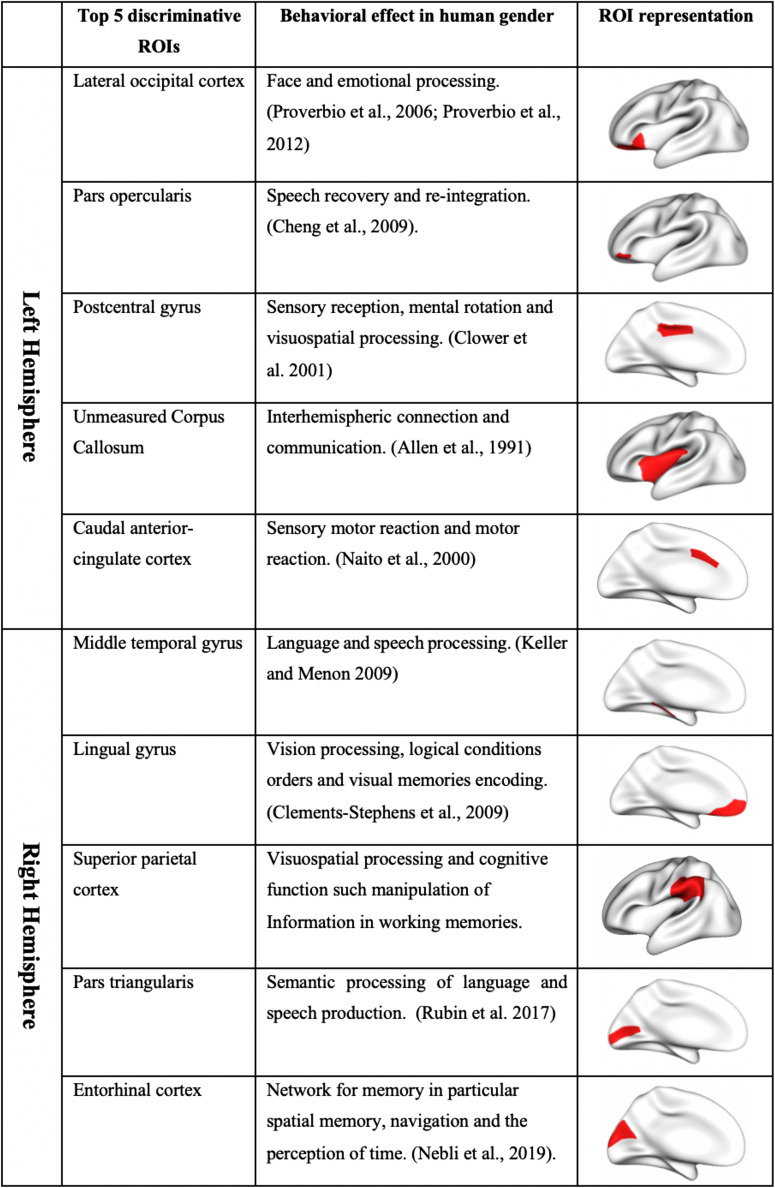
Top 5 discriminative regions of interest (ROIs) in left (LH) and right (RH) hemispheres distinguishing between gender populations revealed by computing the absolute difference between male and female CBTs by MVCF-Net

Our discriminative analysis of the estimated CBTs shows the consistency of our proposed method in relation with MKL technique. By detecting gender-specific biomarkers using both comparative methods, we conclude that our proposed MVCF-Net achieves the highest biomarker reproducibility overlap of the top ROIs distinguishing between male and female CMNs (Tables 3, 4 and Fig. 11). This demonstrates the effectiveness of our method, first in merging complementary information from one population while computing multi-view clustering using manifolds optimization, in which the aligned clusters preserves simultaneously similar and dissimilar traits of the subjects, second in enhancing the distinctive traits between male and female cortical morphological networks while capturing their fingerprinting ROIs.

## Discussion

In this paper, we introduce MVCF-Net, a novel framework for connectional brain template estimation that leverages complementary information offered by multi-view CMNs for a population of multi-view brain networks. Using the estimated CBTs, we identify the top discriminative ROIs distinguishing between genders. First, for each view, MVCF-Net groups similar subjects in the same cluster while separate dissimilar subjects in different clusters. Based on manifold optimization, the clustering process computes the aligned clusters across views to map the subjects to a common space. Then a multi-fusion operation is applied to obtain a representative CBT that captures both shared and differential traits of a population using different views.

### Parameters impacts

The impact of changing the number of clusters *N*_*c*_ on the estimated CBT can be explained by the fact that the k-means clustering algorithm is sensitive to the initial positions of the cluster centroids. As we vary the number of clusters, the total within-cluster variation changes result in different CBTs. We note that the generated CBT depends also on the selected number of nearest neighbors *K*_*n*_. In KNN algorithm, the computation of both pairwise similarity matrix and the Laplacian matrix depends on the value of *K*_*n*_, where a smaller value of *K*_*n*_ can fail to depict a highly heterogeneous multi-peaked distribution of the population whereas a larger value might over-cluster the data and fail to mimic the real distribution. For the selection of the appropriate number of views (cortical attributes), we demonstrate that including four cortical morphological networks will provide the best results in terms of CBT centeredness and representiveness. Constructing the CMNs using all views together achieves the optimal average Frobenius distance and the optimal average Pearson correlation in the left (LH) and right (RH) hemispheres across all methods (Ours and SCA). A combination of morphological attributes has been proven to have better diagnostic performance compared with a single attribute (Yu et al. 2018). This can be explained by the fact that each type of morphological view is derived from a specific cortical measurement will reveal different changes in the morphology of the brain regions. Thus, the constructed CMNs efficiently handle the complexity of the cortical networks and its multivariate interacting effects between the regions which can greatly help in learning a holistic map of the brain connectivity.

### CBT representativeness and centeredness

Our proposed method achieves the best performance in terms of centeredness where the estimated CBTs, derived from male and female populations in both left and right hemispheres, achieve the minimum mean Frobenius distance to all network views (Fig. 9) as well as the highest Pearson correlation when randomly partitioning the data as well as when using the whole data (Fig. 10). These results can be explained by the fact that while SCA integrates heterogeneously the network views lying on different manifolds by merging them directly on a global scale, MVCF-Net learns how to align clusters across views to capture both consistent and differential clusters simultaneously. The correlation between the estimated CBT and all network views in each population is globally consistent across both hemispheres, yet the results between the right and the left hemispheres for gender populations show higher correlations for the left hemisphere. This difference can be explained by the fact that both hemispheres present morphological asymmetry (Witelson and Pallie 1973; Chiron et al. 1995), which generates different CBTs with different centeredness rates.

### CBT discriminability

We demonstrate the discriminative potential of MVCF-Net against SCA in distinguishing between gender populations, where MVCF-Net remarkably achieves the highest matching rate with MKL method of the most 15 discriminative ROIs and the most 20 discriminative ROIs as shown in Table 3, Fig. 11, and Table 4, respectively. These results indicate the effectiveness of our framework in identifying brain regions marking gender differences. This can be explained, first, by the fact that the estimated CBT occupies the minimum distance compared to all subjects in the population, which results in minimizing the inter-subject variability. Second, MVCF-Net is based on multi-view clustering strategy which learns cluster alignment across views to eventually identify both consistent and differential clusters at the same time. Furthermore, MVCF-Net integrates SNF to fuse complementary data lying on different manifolds and avoid dealing with different scales, collection bias and noise in different data types (Wang et al. 2014). Therefore, we believe that MVCF-Net produces more holistic CBT representations for male and female populations, stimulating a deeper understanding of gender difference using multi-view cortical morphological networks.

We display in Table 5 the top 5 discriminative ROIs characterizing the differences between male and female CMNs in the right and left hemispheres. MVCF-Net shows that the top three ROIs distinguishing between genders in the left hemisphere are the lateral occipital cortex, pars opercularis and postcentral gyrus. The lateral occipital cortex is correlated with the control of vision processing specifically facial expression. Our findings are consistent with previous studies, where men showed an asymmetric functioning of visual cortex while decoding faces and expressions, whereas women showed a more bilateral functioning. These results indicate the importance of gender effects in the lateralization of the occipito-temporal response in facial expressions. Other studies supporting our findings, showed a higher activation through a rapid and symmetric of visual time inputs for women rather than men (Proverbio et al. 2006; Proverbio et al. 2012). The reason behind an earlier visual ability is that women have a higher concentration of fibers in the right optic radiation than men (Good et al. 2001). Besides, the pars opercularis region shows an increased volume in young adult females in comparison to males which reflects the high emotional empathic level in women (Cheng et al. 2009). The third most discriminative region, postcentral gyrus, is involved in multiple aspects of sensory processing and sensorimotor integration (Clower et al. 2001) especially in the perception of emotions in facial stimuli (Radua et al. 2010). The study of (Xu et al. 2015) supports our discovery of the postcentral gyrus region as a gender biomarker and showed higher regional homogeneity in females than males. This explains why female generally excel in language (Hyde and Linn 1988; Kimura 1996), facial emotion recognition (Rahman et al. 2004) and emotional memory tasks (Crespo-Facorro et al. 2011).

The fourth most discriminative ROI in LH is the caudal anterior cingulate cortex, which is widely known to be involved in the sensory motor (e.g. motor of reactions) (Naito et al. 2000). While the fifth region corpus callosum has been already demonstrated by a large number of studies to show a sexual dimorphism. This finding can be explained by the difference in the shape of this region between genders, where it was more bulbous shaped in females and more tubular-shaped in males (Allen et al. 1991). Generally, anatomical sex differences such as shape and volume could underlie gender-related differences in behavior and neuropsychological functions.

For the right hemisphere, our method shows that the most three discriminative regions are the middle temporal gyrus, lingual gyrus and superior parietal gyrus. The middle temporal gyrus reveals the difference in the functional organization of the brain activation between male and female brains, where males show a greater ventral stream activation than females. This explains the high mathematical and spatial cognition performances in males (Keller and Menon 2009). The lingual gyrus is responsible for visuospatial processing in mental rotation tasks, where the female brain was shown to use spatial attention and working memory, whereas the male brain uses the visuo-motor network (Clements-Stephens et al. 2009). Other morphological differences in the right occipital lingual gyrus and the right middle temporal gyrus were identified by (Chen et al. 2017), noting that females have significantly increased gray matter concentration rather than male, while males have increased gray matter volume. The third most discriminative ROI, superior parietal gyrus, is correlated with the conscious visual perception of individuals. This focal region showed a difference in brain structure variability between genders which can be explained by gray matter density disparity in the parietal cortex between them.

The fourth most discriminative region in RH is pars triangularis which is important for verbal and language processes. The selection of this region is consistent with (Rubin et al. 2017) study showing the difference of hormone levels in male and female brains responsible for brain system regulation. Compared to women, men showed higher nodal degree and nodal efficiency in pars triangularis. While the entorhinal cortex represents the fifth most discriminative ROI which is consistent with (Nebli and Rekik 2019) finding that this region is considered as a morphological ‘hub’ in CMNs derived from four measurements: maximum principal curvature, mean sulcal depth, mean average curvature and mean cortical thickness. Particularly, the entorhinal cortex might explain the difference in gender behavior and why males and females learn differently.

The difference between the top discriminative regions in the right and left hemispheres are mainly due to the asymmetric nature of the human brain (McGlone 1980; Chiron et al. 1995). This lack of equivalence comes from the difference in cognitive function for each hemisphere called hemisphere lateralization. While the right hemisphere is responsible for the visuospatial processing tasks, which is consistent with our finding about the top discriminative regions for the right part of the brain (e.g. middle temporal gyrus region and lingual gyrus), the left hemisphere is used for linguistic processing and communication which is consistent with our top discriminative regions in the left hemisphere related to facial emotional expressions (Stone et al. 1996). Our results confirm the fact that the top discriminative regions in the right and left hemispheres are different. In fact, (Kovalev et al. 2003; Tranel et al. 2005) demonstrated in their studies the asymmetric influence of gender on the morphological aspects between both hemispheres where male brains were found to be more asymmetric than female. This gender-related effect is noticeable in all brain areas but is most significant in the superior temporal gyrus.

### Study limitations and future recommendations

In summary, we evaluated MVCF-Net, which has the best results in terms of CBT centeredness and representiveness, on morphological connectomic data. Although promising, our method overlooks the topological properties of brain networks when integrating them into a unified CBT. One can integrate topological measures such as degree centrality or betweenness centrality, quantifying the hubness of brain regions in a network, to perverse the population topological properties when estimating the target CBT. We will also tap into the nascent field of graph neural networks (GNNs), which will enable us in an end-to-end manner to learn a CBT without resorting to craftsmanship of an independent data processing steps. We note that our proposed framework is generalizable to different network neuroscience modalities such as functional and structural connectivities, independently. In our future work, we will examine CBTs generated from multimodal brain networks for a more holistic investigation of gender difference at a morphological, functional and structural levels. This will give new insights into how gender-specific brain morphology relates to brain function and structure. Also, we will use different weights for different views (attributes) according to their importance instead of equal weights. As alternative, we propose simultaneous learning of view-specific weights while optimizing the loss function of the multi-view clustering task. This will enable us to identify the most important views in the fusion process and estimation of the gender-specific population-driven CBT.

## Conclusion

In this paper, we proposed a multi-view clustering and fusion network MVCF-Net framework to estimate a well-representative and centered connectional brain template (CBT) for a population of multi-view brain networks. By estimating gender-specific CBTs for male and female cortical morphological networks, respectively, we identified the top cortical ROIs marking gender differences. We demonstrated the outperformance of MVCF-Net in comparison with a state-of-the-art method SCA in terms of (i) centeredness and representativeness compared to all subjects and all views in the population and (ii) discriminability in identifying the most reproducible and discriminative gender connectional markers. By generating a robust and holistic connectional brain map (i.e., CBT) representing a given population, MVCF-Net revealed gender-specific fingerprints using multi-view cortical morphological in relation to behavior, learning, and cognition. In our future work, we will examine how the identified gender cortical morphological markers relate to brain function and structure using multimodal brain networks.

## Data Availability

The open access Brain Genomics Superstruct Project (GSP) (Buckner et al. 2012; Holmes et al. 2015) data that support the findings of this study are available from https://www.nitrc.org/projects/ gspdata website. For reproducibility and comparability, the authors will make available upon request all morphological networks generated based on the four cortical attributes (maximum principal curvature, cortical thickness, sulcal depth, and average curvature) for 698 subjects (308 men and 390 women) following the signed approval by GSP Consortium.
